# Gestational hypothyroxinemia induces ASD-like phenotypes in behavior, proinflammatory markers, and glutamatergic protein expression in mouse offspring of both sexes

**DOI:** 10.3389/fendo.2024.1381180

**Published:** 2024-05-01

**Authors:** Enrique González-Madrid, Ma. Andreina Rangel-Ramírez, María C. Opazo, Luis Méndez, Karen Bohmwald, Susan M. Bueno, Pablo A. González, Alexis M. Kalergis, Claudia A. Riedel

**Affiliations:** ^1^ Laboratorio de Endocrino-inmunología, Departamento de Ciencias Biológicas, Facultad de Ciencias de la Vida, Universidad Andrés Bello, Santiago, Chile; ^2^ Millennium Institute on Immunology and Immunotherapy, Facultad de Ciencias Biológicas, Pontificia Universidad Católica de Chile, Santiago, Chile; ^3^ Facultad de Medicina Veterinaria y Agronomía, Instituto de Ciencias Naturales, Universidad de las Américas, Santiago, Chile; ^4^ Instituto de Ciencias Biomédicas, Facultad de Ciencias de la Salud, Universidad Autónoma de Chile, Santiago, Chile; ^5^ Facultad de Ciencias Biológicas, Pontificia Universidad Católica de Chile, Santiago, Chile; ^6^ Departamento de Endocrinología, Facultad de Medicina, Pontificia Universidad Católica de Chile, Santiago, Chile

**Keywords:** prenatal thyroid function, gestational hypothyroxinemia, neurodevelopment, autism spectrum disorder, behavior, inflammation, NLGN3 and HOMER1 expression

## Abstract

**Background:**

The prevalence of autism spectrum disorder (ASD) has significantly risen in the past three decades, prompting researchers to explore the potential contributions of environmental factors during pregnancy to ASD development. One such factor of interest is gestational hypothyroxinemia (HTX), a frequent condition in pregnancy associated with cognitive impairments in the offspring. While retrospective human studies have linked gestational HTX to autistic traits, the cellular and molecular mechanisms underlying the development of ASD-like phenotypes remain poorly understood. This study used a mouse model of gestational HTX to evaluate ASD-like phenotypes in the offspring.

**Methods:**

To induce gestational HTX, pregnant mice were treated with 2-mercapto-1-methylimidazole (MMI), a thyroid hormones synthesis inhibitor, in the tap-drinking water from embryonic days (E) 10 to E14. A separate group received MMI along with a daily subcutaneous injection of T_4_, while the control group received regular tap water during the entire pregnancy. Female and male offspring underwent assessments for repetitive, anxious, and social behaviors from postnatal day (P) 55 to P64. On P65, mice were euthanized for the evaluation of ASD-related inflammatory markers in blood, spleen, and specific brain regions. Additionally, the expression of glutamatergic proteins (NLGN3 and HOMER1) was analyzed in the prefrontal cortex and hippocampus.

**Results:**

The HTX-offspring exhibited anxious-like behavior, a subordinate state, and impaired social interactions. Subsequently, both female and male HTX-offspring displayed elevated proinflammatory cytokines in blood, including IL-1β, IL-6, IL-17A, and TNF-α, while only males showed reduced levels of IL-10. The spleen of HTX-offspring of both sexes showed increased Th17/Treg ratio and M1-like macrophages. In the prefrontal cortex and hippocampus of male HTX-offspring, elevated levels of IL-17A and reduced IL-10 were observed, accompanied by increased expression of hippocampal NLGN3 and HOMER1. All these observations were compared to those observed in the Control-offspring. Notably, the supplementation with T_4_ during the MMI treatment prevents the development of the observed phenotypes. Correlation analysis revealed an association between maternal T_4_ levels and specific ASD-like outcomes.

**Discussion:**

This study validates human observations, demonstrating for the first time that gestational HTX induces ASD-like phenotypes in the offspring, highlighting the need of monitoring thyroid function during pregnancy.

## 1 Introduction

Maternal thyroid hormones (THs) - L-3,5,3’,5’-tetraiodothyronine (T_4_) and L-3,5,3’-triiodothyronine (T_3_) - play pivotal roles in fetal growth and development (1, 2). The developing fetus completely relies on maternal THs until approximately the 16^th^ to 20^th^ week of gestation, when its own thyroid gland matures and commences hormone synthesis (3, 4). This dependency arises from maternal thyroid adaptations in pregnancy, which ensure adequate TH levels to fulfill the mother and fetal metabolic demands (3, 4). The transplacental transfer of T_4_ from the maternal circulation to the fetus is a particular critical event, given its greater propensity to cross the placental barrier compared to the biologically active T_3_. This facilitates the direct utilization of T_4_ by fetal tissues or its conversion into T_3_ (5, 6). The increased maternal thyroid function in pregnancy also raises the gland susceptibility to dysfunction (7). In this context, gestational hypothyroxinemia (HTX) is an asymptomatic and highly frequent condition in early pregnancy, clinically defined by a reduction in T_4_ (below the 5th percentile), while maintaining normal levels of T_3_ and thyroid-stimulating hormone (TSH) in blood (8–10). Gestational HTX has been an overlooked condition given its asymptomatic nature, nonetheless, is associated with neurocognitive impairments in the offspring, including lower intelligence quotient (IQ), auditory and motor difficulties, as well as an increased risk of attention deficit hyperactivity disorder (ADHD) in children (11–16). Interestingly, retrospective studies in humans have reported that 5-to-8-year-old offspring gestated under HTX in the first trimester of pregnancy have a 2-to-4-fold increased likelihood of having autistic traits in behavior (17, 18).

Autism spectrum disorder (ASD) is defined as a sex-biased neurodevelopmental disorder (referring to gender-dependent differences in the occurrence or impact of ASD), characterized by social impairments and restricted repetitive patterns of interest and behavior (19). ASD is a heterogenous condition displaying a broad spectrum of cognitive abilities that often coexists with a spectrum of comorbidities, including epilepsy, feeding difficulties, constipation, attention-deficit hyperactivity disorder (ADHD), anxiety, and depression (19). The World Health Organization (WHO) recognizes ASD as a widespread condition, affecting approximately 1% of the global population (20). Over the past two decades, there has been a notable increase in its prevalence, shifting from 1 in 150 to 1 in 36 in the US. This rise is largely attributed to heightened awareness and advancements in early diagnosis methodologies (20). However, epidemiological and scientific studies indicate that ASD results from intricate interactions involving both genetic predispositions and environmental influences (21–24). In this context, exposure to environmental factors during pregnancy has been identified as potential contributors to the prenatal induction of ASD, including environmental pollution (25–27), the use of neuropsychiatric drugs or teratogens (28, 29), parental aging (30), and maternal inflammation (31). In addition to known thyroid disruptors such as perchlorate and pesticides, it has been demonstrated that these environmental factors that can also impair the adequate functioning of the maternal thyroid gland during pregnancy are associated with the emergence of ASD-like phenotypes in the offspring (32–34).

Mouse models have been essential in studying ASD, reflecting some aspects of the disorder found in humans (35). These models are typically assessed based on behaviors like repetitive actions, anxiety, and social interaction (36). Despite the valuable contributions of these models in uncovering the biological mechanisms of autism, inconsistencies in behaviors are frequently noted, reflecting the variations observed in humans (37–39).

Apart from the behavioral aspect, it has been reported that the expression of synaptic scaffolding proteins of glutamatergic neurons such as Homer protein homolog 1 (HOMER1) and Neuroligin 3 (NLGN3) are altered in the prefrontal cortex (PFC) and hippocampus of animal models with ASD like phenotype (40, 41). This is the case described in adult rats prenatally exposed to valproic acid (VPA) that also exhibit ASD-like behaviors (42). Moreover, both point mutations and altered expression of NLGN3 in mice have been shown to induce defective synaptic pruning and impaired hippocampal circuit-specific organization underlying the ASD-like phenotype (43, 44).

ASD-like alterations extend beyond the central nervous system. Immune dysregulation has been observed in both adult ASD patients and ASD-like rodent models assessed during adulthood (45, 46). Increased serum concentration of proinflammatory cytokines like interleukin (IL)-1β, IL-6, IL-17A, and TNF-α have been detected and are hypothesized to contribute to ASD pathophysiology. Consistently, anti-inflammatory cytokines like IL-10 and TGF-β1 are typically lower in ASD patients (45, 46). Additionally, elevated concentration of IL-17A in specific brain regions, which is considered as a neuroinflammatory-like trait, is associated with alterations in social cognition and appears to be a signature outcome of ASD physiopathology according to animal models displaying ASD-like behaviors (47). Moreover, ASD-related inflammatory profile can be accompanied by persistent dysregulation of both innate and adaptive immune response, as indicated by murine models. For instance, both the inbred mouse strain BTBR T^+^Itpr3*^tf/J^* (murine model of idiopathic ASD) and the adult mice gestated under maternal inflammation activation (MIA) model display increased population of macrophages, natural killers (NKs), and T cell populations in the spleen as compared to *wild-type* C57BL/6 mice (48–50).

Despite human retrospective studies associating gestational HTX with ASD behavioral traits in the offspring, this notion has not been validated yet, at the same time that ASD-like cellular and molecular alterations have not been evaluated in this offspring. Therefore, by using a mouse model, this study aimed to establish a causal relationship between gestational HTX and ASD-like alterations in behavior, inflammatory biomarkers, and levels of specific glutamatergic proteins in the offspring of both sexes.

## 2 Materials and methods

### 2.1 Animals

C57BL/6 mice obtained from The Jackson Laboratory, Bar Harbor, ME, USA were housed in cages with water, bedding, and chow (Prolab, RMH 3000) under standard conditions of a 12-hour light/dark cycle (lights on at 8:00 AM) and a temperature of 22°C at the animal facility of the Facultad de Ciencias de la Vida, Universidad Andrés Bello (Santiago, Chile). All experimental procedures and care were performed according to current regulations and guidelines of the Animal Welfare Committee of the Facultad de Ciencias de la Vida (Bioethics approval certificate number 012/2021) and Agencia Nacional de Investigación y Desarrollo (ANID). Daily supervision of mice was performed by a veterinarian. Mice were euthanized by inhaling 5% isoflurane/O_2_ according to AVMA guidelines (51).

### 2.2 Induction of gestational hypothyroxinemia

Gestational HTX was induced in pregnant mice according to previous reports (52–55). Six- to eight-week-old C57BL/6 mice were mated in a proportion of 2 females and 1 male. The next day, a vaginal smear was collected and analyzed to search for spermatozoa. Mice with a positive smear were considered pregnant, and that day was referred to as embryonic day 1 (E1). Each dam was individually placed in a cage. On E10, pregnant mice were randomly distributed into three groups (N = 7 pregnant mice per group). The first group received regular tap water during the entire pregnancy (untreated dams); therefore, gestation was conducted under euthyroid conditions. The second group received 0.025% w/v of 2-mercapto-1-methylimidazole (methimazole, MMI) (M8506, Sigma-Aldrich, USA) in the drinking water from E10 to E14 (MMI-treated dams). To reverse the MMI-induced phenotypes, the third group was treated with 0.025% w/v MMI along with a daily subcutaneous injection of 25 μg/kg of T_4_ dissolved in PBS from E10 to E14 (MMI+T_4_-treated dams) (52–55). This treatment aimed to demonstrate that the observed effects induced by MMI are due to maternal T_4_ reduction and not a side effect of MMI. Untreated and MMI-treated dams did not receive a vehicle intraperitoneal injection to avoid the related stress of a daily injection. Both the MMI and MMI+T_4_ treatments were administered daily from E10 to E14 at 9:00 AM. The water for untreated dams was also renewed during E10 to E14. The bottles containing MMI solution were prepared daily with fresh tap water and protected from light. Consequently, the experimental groups of this work were the respective progenies named after the mother’s treatment: Control-offspring, HTX-offspring, and HTX+T_4_-offspring.

### 2.3 Determination of thyroid hormones

To verify the correct induction of gestational HTX, blood samples were collected from the facial vein of each pregnant mouse on E14 and total (t)THs and TSH were quantified. Additionally, a blood sample was also obtained from each adult mouse on postnatal day 50 (P50) to evaluate thyroid function. Serum was separated from the blood samples by centrifugation at 1,000 xg for 15 min at 4°C. Levels of tT_3_ and TSH were quantified by sandwich ELISA using mouse CUSABIO® ELISA kits (catalog N°E05086m and N°E05116m, respectively) following the manufacturer’s instructions. Concurrently, tT_4_ serum levels were determined by chemiluminescence in an external certified veterinary laboratory (LQCE, Santiago de Chile). After quantification, the tT_4_ range for MMI-treated dams was established at [0.1 – 1.9] (μg/dL), whereas the range of tT_4_ in the untreated dams was [2.2 – 4.5] (μg/dL). Since the administration of tT_4_ during MMI treatment is expected to restore their levels and reverse the induced phenotype, the accepted tT_4_ range in the MMI+T_4_-treated dams was set equal to that of the untreated group ([2.2 – 4.5] (μg/dL)) (52–55).

### 2.4 Behavioral testing

Control, HTX, and HTX+T_4_ offspring were weaned on P28. To mitigate the impact of the “litter effect”, experimental groups were formed by randomly selecting 2-3 mice of each sex from each litter. Consequently, each group comprised 30 mice, conformed by 15 females and 15 males. Mice of the same sex were housed together in cages (4-5 mice per cage) from weaning until behavioral assessment. Behavioral testing was conducted during adulthood (from P55 to P64), specifically during the dark phase of the circadian cycle (between 9.00 AM and 2.00 PM), consistent with a previous report of behavioral assessment in mice (56). The sequence of behavioral testing was arranged as follows: marble burying (MB) on P55, elevated plus maze (EPM) on P56, tube dominance test from P57 to P63 (considering training and confrontation steps), and three-chamber social preference test on P64. The behavioral performance of each mouse in the EPM, tube dominance, and three-chamber tests was conducted under 60 decibels (dB) of white noise and recorded using an FHD 1080p/30 fps camera. Data were manually scored and analyzed by three trained investigators blinded to each experimental group.

### 2.5 Marble burying test

MB was performed to analyze repetitive/persistent behavior (57, 58). Each mouse was placed inside the testing cage (arena size: 24 cm x 17.2 cm, wood chips bedding depth: 5 cm) containing 12 marbles arranged in four columns of three marbles at equidistant distances. After 30 min of exploration, each mouse was returned to its respective cage. The number of buried marbles was counted, considering them buried when 2/3 of their volume was concealed within the wood chips.

### 2.6 Elevated plus maze test

The elevated plus maze (EPM) test was employed to analyze anxious-like behavior (59, 60). Each mouse was placed in the center of a suspended cross-shaped maze consisting of two open and two closed arms (25 cm long x 7 cm wide x 24.5 cm high walls of closed arms). After 5 minutes of exploration, each mouse was returned to its respective cage. The number of entries into both the open and closed arms, as well as the time spent in each arm, were recorded. An entrance was only considered valid when all four paws of the mouse crossed the boundary of the maze center into an open or closed arm.

### 2.7 Tube dominance test

The tube dominance test was used to assess aggressive social interaction (61). A hollowed transparent Plexiglas tube measuring 30 cm in length and 2.5 cm in diameter was used. Two mice from distinct experimental group were introduced to the tube from opposite directions. The critical point occurs when both mice meet in the middle of the tube, and one must retreat to exit, thereby revealing a dominant or subordinate status based on the species hierarchy theory. The training step was conducted over three consecutive days (from P57 to P59) with the goal of teaching each mouse that crossing the tube is the only way to exit without backing up. Subsequently, the testing stage took place over four consecutive days (from P60 to P63). During the confrontation stage, mice from the Control-offspring were paired with those from the HTX-offspring group, the HTX-offspring mice were paired with the HTX+T_4_-offspring mice, and the Control-offspring mice were paired with the HTX+T_4_-offspring mice in a pairwise fashion. Confrontations were conducted between mice of the same sex, with pairs randomly chosen and confronted three times per day, rotating the initial entry position to minimize bias. After each confrontation, mice were returned to their respective cages to reduce the immediate impact of recent winning or losing. The number of wins for each mouse was recorded, and the total number of wins after four days was used as an indicator of their social status, distinguishing between dominant or subordinate.

### 2.8 Three-chamber social preference test

The three-chamber social preference test was conducted to assess social interaction abilities (62). Mice were first habituated per 1 h to the three-chamber device (a plexiglass box of 102 cm length x 47 cm width x 45 cm height). The three-chambered apparatus consisted of two lateral compartments, each containing one inverted wire cup, along with one empty central compartment. During the pre-test, each mouse was allowed to acclimate to the setup for 10 minutes. Then, the first phase of the test was initiated. An inanimate object was placed under one cup (named “nonsocial stimulus”), while an unfamiliar mouse, matched in terms of age and sex, was placed under the other cup (named “social stimulus”). Each mouse was then positioned in the central compartment of the three-chamber device and allowed to transit between the nonsocial and social compartments for 10 minutes. After that time, mice were returned to their respective cages. In the second phase of the test, the nonsocial stimulus was replaced with a second unfamiliar, age- and sex-matched mouse (also named “*de novo* stimulus” or “Stranger 2”), while maintaining the first strange mouse now renamed “Stranger 1”. Each mouse was allowed to transit between the Stranger 1 and Stranger 2 compartments for 10 min. The following parameters were registered: the time that each mouse spent in the social, nonsocial, and *de novo* compartments, the time of direct interaction with the social, nonsocial, and *de novo* stimulus, and the number of entrances to each compartment. Direct interaction was defined as the presence of the mouse within a diameter of 5 cm around each cup. Similar to the EPM test, an entry was considered when all four legs of the mouse crossed the center of the chamber toward one of the lateral compartments.

### 2.9 Cytokine determination by sandwich ELISA

On the day following the last behavioral test (P65), the Control, HTX, and HTX+T_4_ offspring were euthanized to obtain the blood, brain, and spleen. Blood was collected via cardiac puncture and allowed to clot at room temperature for 25-30 minutes. Afterward, serum was isolated by centrifugation (1,000 xg, 15 minutes at 4°C), aliquoted, and stored at -80°C until further analysis. Brains were dissected to isolate the prefrontal cortex (PFC) and hippocampus from the telencephalon, following a standardized mouse brain dissection protocol (63). Both tissues were homogenized in radioimmunoprecipitation assay (RIPA) buffer (50 mM Tris-HCl pH 8.0, 150 mM NaCl, 0.1% SDS, 0.5% sodium deoxycholate, 0.1% Triton X-100) supplemented with phosphatase and protease inhibitors (1 mM Na_3_VO_4_, 1 mM NaF, 30 mM sodium pyrophosphate, and 1 mM PMSF). After a 30-minute incubation on ice, the homogenates were centrifuged at 15,000 xg for 15 minutes at 4°C. Total protein quantification from the supernatants was performed using the BCA method with a BSA standard curve (PierceTM, BCA™ 23225). Cytokines were quantified in serum samples of 100 μL and in brain tissue portions (0.5 mg of protein) using sandwich ELISA kits for specific cytokines: IL-1β (BioLegend, 432601), IL-6 (BioLegend, 431307), IL-17A (BioLegend, 432501), IL-10 (BioLegend, 555252), and TNF-α (BioLegend, 430901), following the manufacturer’s instructions. Detection was conducted using a microplate spectrophotometer (Epoch™). Serum cytokine concentrations were normalized to a volume of 100 μL, while cytokine levels in brain tissue portions were normalized to the amount of protein used for these measurements. Results are presented as pg/mL for cytokines in serum and as pg/mg for normalized brain-derived cytokines.

### 2.10 Detection of NLGN3 and HOMER1 by western blot

The relative expression of the glutamatergic proteins NLGN3 (94 kDa) and HOMER1 (40 kDa) in the PFC and hippocampus was evaluated by western blot. Initially, 20 µg of protein from both tissues was denatured at 95°C for 5 min and then separated on a 12.5% SDS-PAGE gel at 100 V for 120 min. The PageRuler Plus Prestained Protein Ladders of 10-250 kDa (Thermo Scientific™ 26619) and 10-180 kDa (Thermo Scientific™ 26616) were used to estimate the molecular weight of NLGN3 and HOMER1, respectively, and to monitor the progress of electrophoretic separation and electrotransference. Proteins were transferred onto nitrocellulose membranes (Thermo Scientific) for 1 h at 100 V on ice. Thereafter, membranes were blocked with 5% nonfat milk dissolved in Tris buffered saline with 0.1% Tween-20 (TBS-T, pH 7.6) for 1h at R.T with constant shaking. Membranes were separately probed with the primary antibodies anti-NLGN3 (2 μg/mL) (ab192880) and anti-HOMER1 (0,75 μg/mL) (ab211415), both prepared in 5% nonfat milk in TBS-T, overnight at 4°C and constant rotation. Moreover, α-tubulin (1 μg/mL) (ab6046) was used as a loading control. On the next day, membranes were washed (3 times, 5 min each) using TBS-T and then incubated with the secondary antibody anti-rabbit HRP-conjugated (1 μg/mL) (Bethyl A120-101P) in 5% nonfat milk in TBS-T for 1h at R.T and constant rotation. Membranes were washed (3 times, 5 min each) using TBS-T and incubated with ECL detection substrate (Thermo ScientificTM 34076) for 5 min. Bands were visualized and images were recorded using the Alliance Q9 Advanced chemiluminescence and spectral fluorescence imaging system (UVITEC Cambridge). Band intensities were analyzed using ImageJ software (NIH, Bethesda, MD, USA) and the relative expression of NLGN3 and HOMER1 was normalized to α-tubulin.

### 2.11 Total splenocytes isolation

Spleens were separately placed in fresh RPMI-1640 medium supplemented with 10% fetal bovine serum (FBS) and then homogenized through a 70 µm cell strainer. Cell suspensions were centrifuged at 460 xg for 5 min at 4°C and then treated for 5 min at R.T with ammonium-chloride-potassium buffer (ACK, pH 7.2) to lyse erythrocytes. After two washes with fresh 1X PBS (5 mL each), total splenic leukocytes (also called splenocytes) were resuspended in 1 mL of PEB buffer (PBS 1X, 0.5% BSA, 2 mM EDTA), and counted using the Countess 3 Automated Cell Counters equipment (Thermo Fisher Scientific) using the trypan blue exclusion test of cell viability analysis (64).

### 2.12 Immune cell assessment by flow cytometry

Adaptative and myeloid immune cell populations from the splenocytes were analyzed by flow cytometry. A portion of 1.0×10^7^ splenocytes were stimulated *in vitro* with phorbol myristate acetate (PMA; 5 ng/mL), ionomycin (500 ng/mL), and brefeldin-A (BFA, 10 μg/mL) for 5 h at 37°C and 5% CO_2_. Cells were harvested by centrifugation (600 xg, 5 min, 4°C) and then incubated with the following antibodies for 45 min at 4°C to assess Treg and Th17 populations: anti-CD4-BUV395 (BD 563790), anti-CD25-PeCy7 (BioLegend 102016), and anti-IL-17A-APCCy7 (BD 560821). Cells were washed and fixed with 2% formaldehyde in PBS for 15 min at R.T. After washing with PEB buffer, the cells were permeabilized using buffer IV (BD Phosflow 560746) during 15 minutes at 4°C. Cells were then washed and incubated with anti-FOXP3-Pe (Invitrogen 12-5773-82) and anti-RORγt-BV421 (BD 562894) during 45 min at 4°C. Antibodies were used at 0.2 mg/mL. Cells were washed and fluorescent beads (Thermo Fisher C36950) were added to each sample at a concentration of 667 beads/mL to determine the absolute number of events of interest. Absolute numbers of Treg and Th17 cells were also used to determine the Th17/Treg ratio as previously reported (65). Finally, 300,000 events per sample were recorded using the BD LSRFFortessa X-20 cytometer from the Facultad de Ciencias Biológicas, Pontificia Universidad Católica de Chile. FlowJo software (version 10.8) was used to analyze the immune cell populations. Concurrently, to analyze myeloid immune cell populations, a portion of 1.0×10^7^ splenocytes were directly stained with the following antibodies to analyze myeloid immune cell populations: anti-CD45-FITC (BioLegend 109806), anti-CD3-PercpCy5.5 (BioLegend 100218), anti-CD11b-PeCy7 (Invitrogen 25-0112-82), anti-CD49b-APC (BioLegend 103515), and anti-NK1.1-BV605 (BioLegend 108739) for Natural killers (NKs); anti-CD45-FITC (BioLegend 109806), anti-F4/80-BUV395 (BD 565614), anti-CD80-BV605 (BioLegend 104729), and anti-CD68-PeCy7 (BioLegend 137015) for M1-like macrophages; and anti-CD45-FITC (BioLegend 109806), anti-F4/80-BUV395 (BD 565614), anti-CD163-APC (BioLegend 156705), and anti-CD206-BV421 (BioLegend 141717) for M2-like macrophages, during 45 min at 4°C and darkness. All antibodies were used at 0.2 mg/mL. Cells were washed and analyzed by flow cytometry as previously described.

### 2.13 Statistical analyses

Sample sizes were determined using G*Power 3.1.9.7 software, based on a previous report (66). For pregnant mice groups, one-way ANOVA in F tests was conducted, with an effect size set to 0.80, α set to 0.05, and a test power set to 0.80, and considering 3 groups. This calculation yielded a total sample size of 21, with 7 mice allocated to each group. For experimental groups (Control-offspring, HTX-offspring, and HTX+T_4_-offspring), sample size was determined using a mixed-effects model in F test ANOVA, with an effect size set to 0.5 (effect size specification according to Cohen, 1988 (67)), α set to 0.05, a test power set to 0.99, number of measurements set to 41 (comprising all independent measurements obtained from each evaluated parameter in this study) and considering 3 groups. This resulted in a total sample size of 90, distributing 30 mice per experimental group, and separating 15 mice per sex. This allocation of mice for behavioral assessment and subsequent molecular analysis on the same mice aligns with the reduction and refinement principles of the 3 Rs in bioethics (68). Data analysis and statistical tests were performed using Prism 9.0.2 software (GraphPad, Inc). Thyroid hormone levels were compared among pregnant mice groups using *one-way* ANOVA followed by Tukey’s post-hoc test. To conduct multiple comparisons between experimental groups and both sexes for each parameter evaluated in this study, while mitigating the impact of the “litter effect”, a mixed-effects model was employed followed by Tukey’s post-hoc test. This approach enhances the reliability and accuracy of the results, ensuring robust statistical analysis (69). Pearson correlation analysis was applied to associate maternal T_4_ levels with all outcomes evaluated in the offspring. The results are presented as the mean ± standard error of the mean (SEM). Significance was established at *p*<0.05. *p* values in figures and text are presented as follows: *p<0.05, **p<0.01, ***p<0.001, and ****p<0.0001.

## 3 Results

### 3.1 Methimazole treatment during E10 and E14 induces hypothyroxinemia in dams

**Figure 1:**
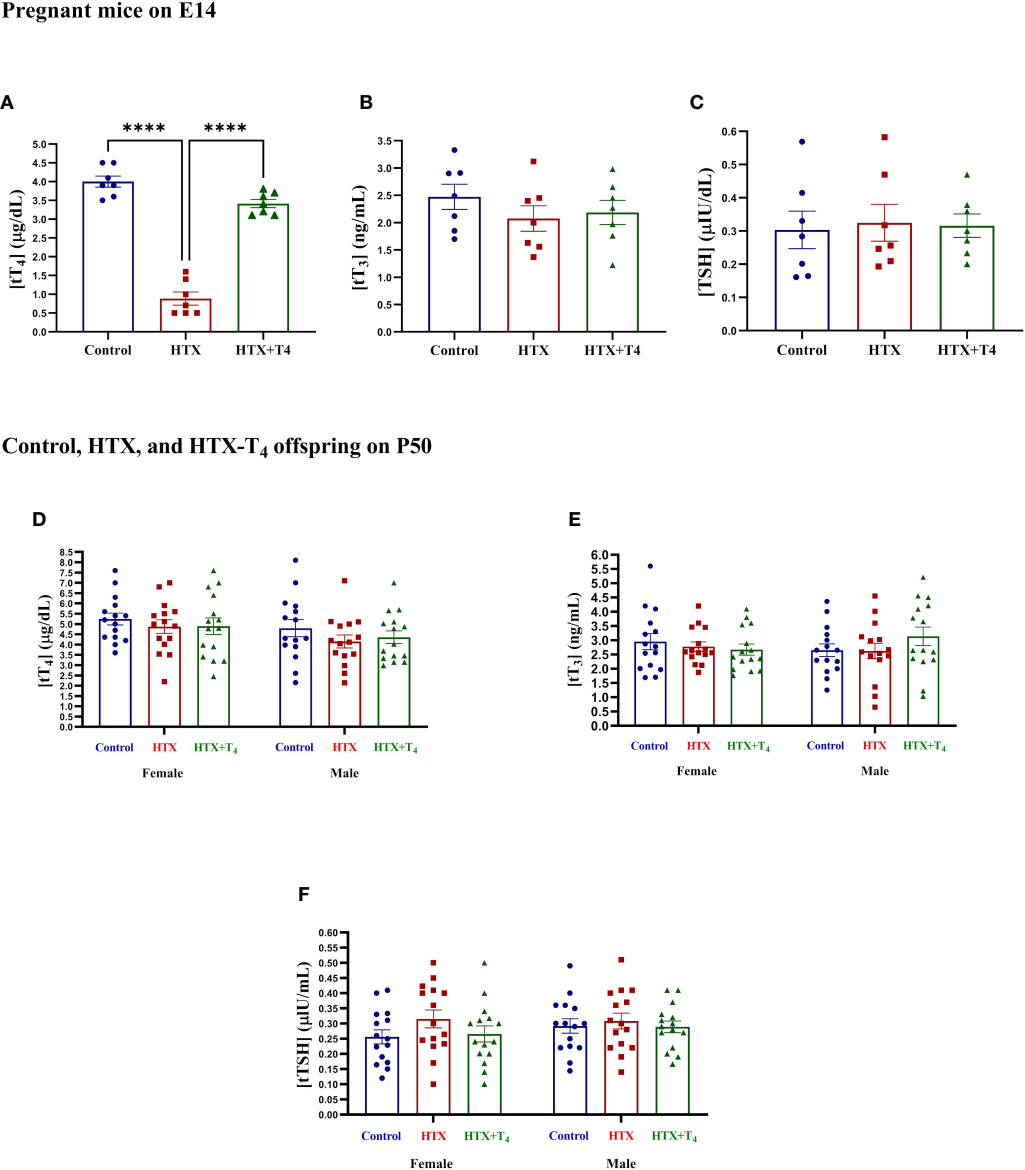
Methimazole treatment induces gestational hypothyroxinemia in pregnant mice, while adult offspring remain euthyroid. Levels of total TH and TSH were measured in blood samples obtained from the facial vein of Control, HTX, and HTX+T_4_ dams on E14 and plotted at **(A)** tT_4_, **(B)** tT_3_, and **(C)** TSH. Furthermore, the same hormones were quantified in both female and male offspring from the three experimental groups on P50 **(D-F)** (see Materials and Methods section). **(A-C)** N = 7 pregnant mice per group. **(D-F)** N = 15 per group and sex. Data are presented as mean ± S.E.M. Differences between pregnant mice groups **(A-C)** were analyzed by *one-way* ANOVA and Tukey’s post-hoc. (****p<0.0001). Multiple comparisons between experimental groups and both sexes **(D-F)** were analyzed by Mixed-effects model and Tukey’s post-hoc. Control-offspring: blue circles, HTX-offspring: red squares, and HTX+T_4_-offspring: green triangles.

Gestational HTX was induced in pregnant mice by administering 0.025% w/v of methimazole (MMI) through the tap drinking water from embryonic day 10 (E10) to E14 (refer to Materials and methods). The E10 to E14 timeframe for MMI treatment was chosen because it is comparable to the first trimester of gestation in human brain development (70). MMI acts as an allosteric inhibitor of the thyroid peroxidase enzyme, hindering the coupling of molecular iodine to the tyrosine residues in the thyroglobulin molecule, thereby inhibiting the TH synthesis (52–55). Several reports have shown that short-term administration of this drug at this concentration induces gestational HTX rather than gestational hypothyroidism (52–55). To confirm the successful induction of gestational HTX in dams, the levels of tT_4_, tT_3_, and TSH were quantified from serum samples obtained on E14. Results are shown in **Figure 1**. The levels of tT_4_ in the MMI-treated dams were reduced compared to untreated dams, however, this decrease was reversed by the addition of T_4_ during the MMI administration (**Figure 1A**). Additionally, the levels of tT_3_ (**Figure 1B**) and TSH (**Figure 1C**) remained similar among untreated, MMI-treated, and MMI+T_4_-treated dams. The exclusive reduction in tT_4_ confirmed the successful induction of HTX. Additionally, tT_4_, tT_3_, and TSH were quantified in the Control, HTX, and HTX+T_4_ offspring of both sexes on P50. The results indicate that the levels of tT_4_ (**Figure 1D**) tT_3_ (**Figure 1E**) and TSH (**Figure 1F**) were unchanged across the groups and sexes, indicating that the MMI treatment did not impact the offspring’s thyroid function in adulthood.

### 3.2 Gestational HTX induces ASD-like behavioral alterations in adult offspring of both sexes

Retrospective studies in humans have associated gestational HTX with autistic behavioral traits in the offspring (17, 18). However, comprehensive causal studies, along with an investigation into the cellular and molecular mechanisms underlying these ASD-like manifestations in the offspring, have yet to be conducted. Therefore, using a mouse model, the primary aim of this study was to validate the notion that gestational HTX leads to the development of ASD-like behavioral alterations in the offspring. Furthermore, considering potential sex-specific effects, both females and males were included in the exploration of these outcomes. For this purpose, four behavioral tests were employed to evaluate repetitive behavior, anxious-like behavior, and social interaction abilities in the HTX-gestated offspring of both sexes and compare their performance with that of Control and HTX+T_4_ offspring of both sexes. Behavioral testing was performed during adulthood given it is known that the brain reaches maturity at synaptic level and behavior-relevant areas, enabling a more precise investigation of ASD-like associated cognitive functions (70). Data are provided in **Supplementary Table 1**.

**Figure 2:**
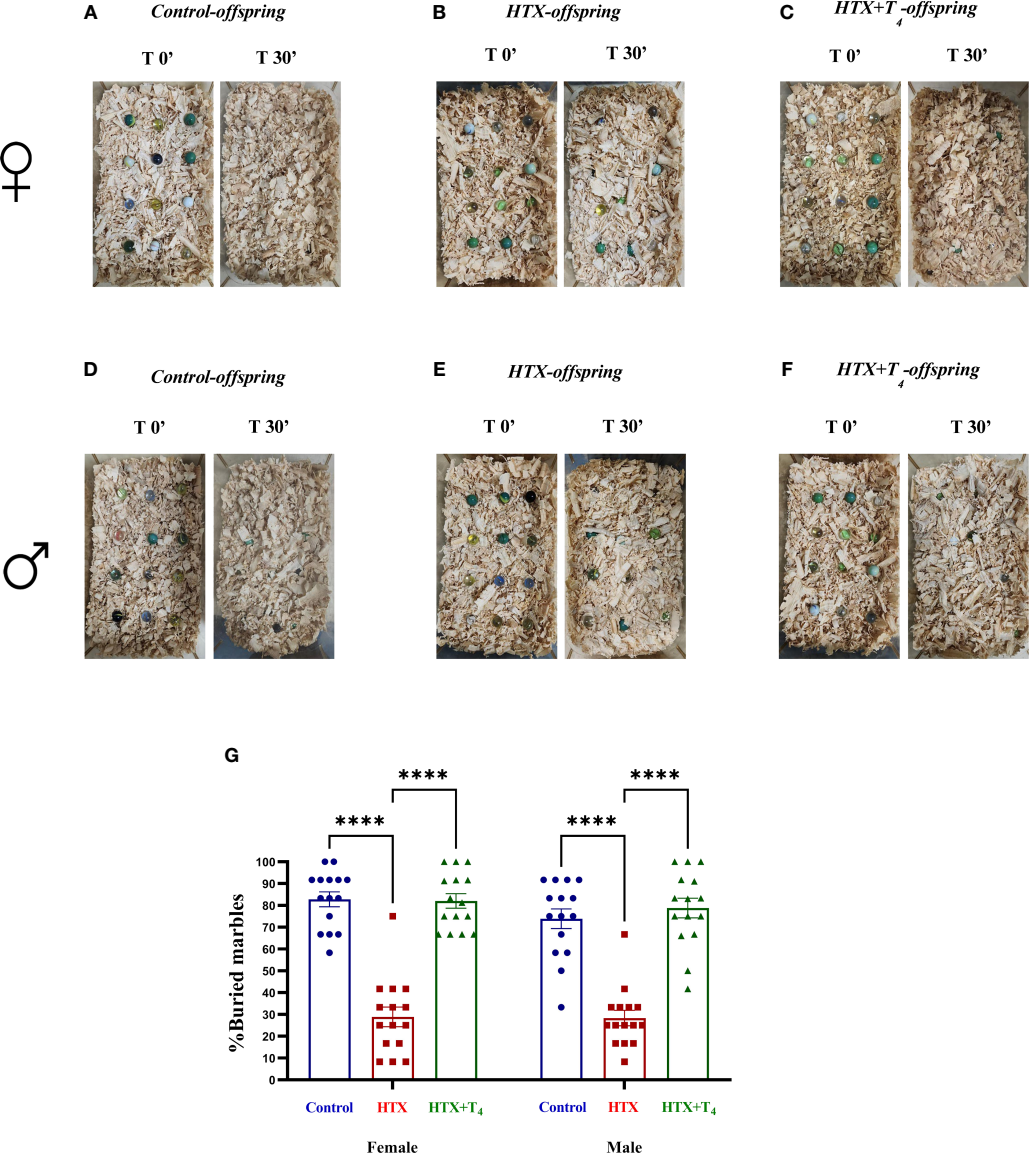
The HTX-gestated offspring of both sexes buried fewer marbles compared to the Control and HTX+T_4_ offspring. To assess repetitive behavior, adult offspring from the three experimental groups underwent the marble burying test on P55. The number of buried marbles was recorded, and the average count per experimental group was calculated. Pictures of the marbles at time 0’ and 30’ of the male **(A-C)** and female **(D-F)** experimental groups are illustrated. **(G)** The graph shows the percentage of buried marbles. N = 15 per group and sex. Data are presented as mean ± S.E.M. Multiple comparisons between experimental groups and both sexes were analyzed by Mixed-effects model and Tukey’s post-hoc. (****p<0.0001). Control-offspring: blue circles, HTX-offspring: red squares, and HTX+T_4_-offspring: green triangles.

First, repetitive behavior was assessed using the marble burying test, where increased number of buried marbles compared to the control group indicates ASD-like repetitive behavior (57, 58). Female and male Control, HTX, and HTX+T_4_ offspring were individually placed in the testing cage containing twelve glass marbles for 30 min to evaluate their burying behavior. After that time, mice were returned to their respective cages and the number of buried marbles was counted (see Materials and methods). The percentages of buried marbles are shown in **Supplementary Table 1.1**. The representative images illustrate the buried marbles of both the female Control (**Figure 2A**), HTX (**Figure 2B**), and HTX+T_4_ (**Figure 2C**) offspring, as well as the male Control (**Figure 2D**), HTX (**Figure 2E**), and HTX+T_4_ (**Figure 2F**) offspring. Quantified data indicate that both female and male HTX-offspring significantly buried fewer marbles than the Control and HTX+T_4_-offspring (**Figure 2G**, **Supplementary Table 1-1.1**), suggesting the HTX-gestated offspring do not manifest a repetitive ASD-like behavior.

**Figure 3:**
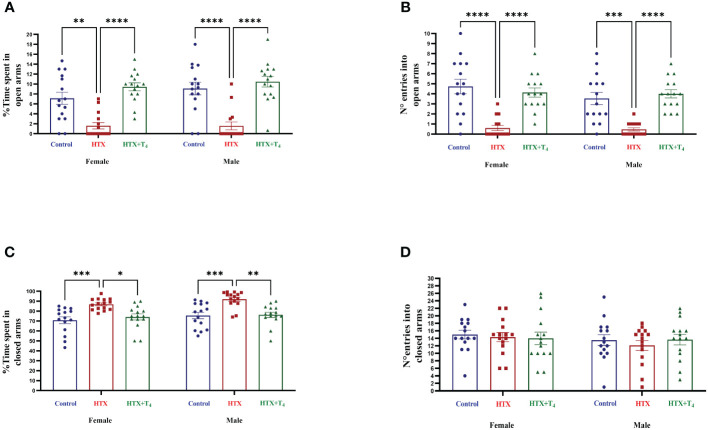
Female and male HTX-offspring exhibited anxious-like behavior. To assess anxious-like behavior, both male and female offspring from the three experimental groups were subjected to the elevated plus maze test on P56. **(A)** Percentage of time spent in open arms, **(B)** Number of entrances into the open arms, **(C)** Percentage of time spent in closed arms, and **(D)** Number of entrances into the closed arms. N = 15 per group and sex. Data are presented as mean ± S.E.M. Multiple comparisons between experimental groups and both sexes were analyzed by Mixed-effects model and Tukey’s post-hoc. (*p<0.05, **p<0.01, ***p<0,001, ****p< 0.0001). Control-offspring: blue circles, HTX-offspring: red squares, and HTX+T_4_-offspring: green triangles.

Since anxiety is a widespread comorbid condition in ASD (71), the second evaluated aspect was the anxious-like behavior through the elevated plus maze (EPM) test (59, 60). Mice were individually placed in the cross-shaped maze and the time spent in both the open and closed arms and the number of entrances were registered (see Materials and methods). Results are shown in **Figure 3** and **Supplementary Table 1-1.2**. The findings indicate that both female and male HTX-offspring spent considerably less time exploring the open arms (**Figure 3A**, **Supplementary Table 1-1.2a**), which was consistent with a lower number of entries into the open arms (**Figure 3B**, **Supplementary Table 1-1.2b**), compared to the Control and HTX+T_4_ offspring. Conversely, both female and male HTX-offspring spent significantly more time hiding in the closed arms, compared to the Control and HTX+T_4_ offspring (**Figure 3C**, **Supplementary Table 1-1.2c**). No differences in the number of entrances to the closed arms were detected across experimental groups and sexes (**Figure 3D**, **Supplementary Table 1-1.2d**). Considering that when mice spending more time hiding in the closed arms of the maze and less exposure to open spaces points to anxious-like behavior, these results suggest that the HTX-offspring manifests a behavior indicative of anxiety.

**Figure 4:**
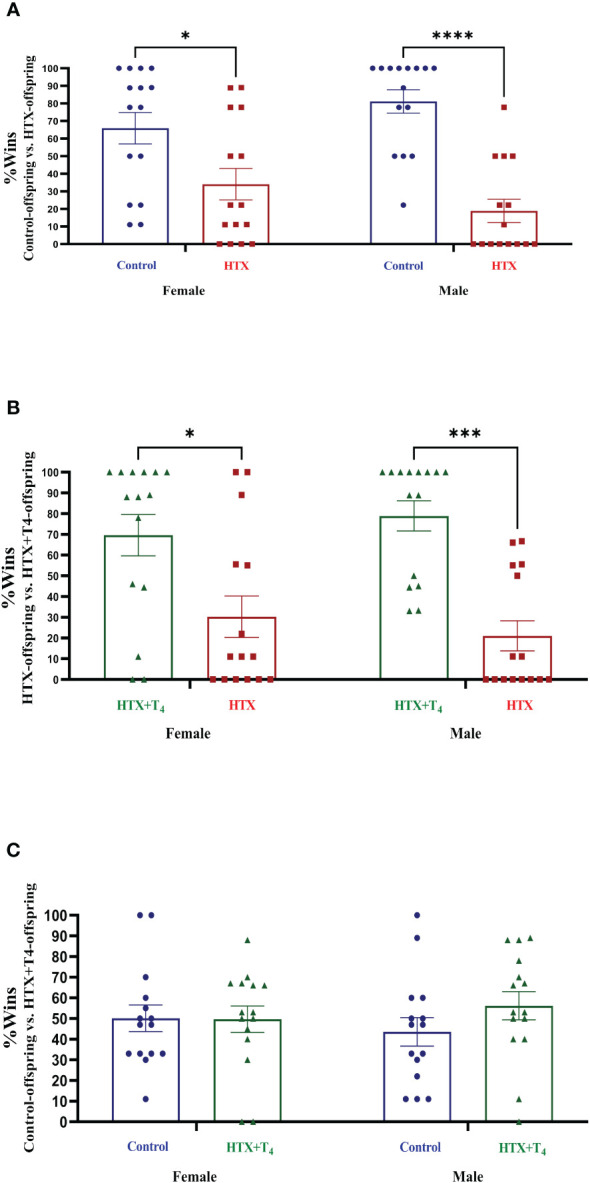
The HTX-gestated offspring of both sexes exhibited a subordinate state in the tube dominance test. Offspring from the three experimental groups were submitted to the tube dominance test (from P57 to P63) to study their socially aggressive behavior (details in Materials and methods section). Confrontations were arranged as follows: **(A)** Control-offspring vs. HTX-offspring, **(B)** HTX-offspring vs. HTX+T_4_-offspring, and **(C)** Control-offspring vs. HTX+T_4_-offspring. The percentage of wins was plotted. N = 15 per experimental group and sex. Data are presented as mean ± S.E.M. Multiple comparisons between experimental groups and both sexes were analyzed by Mixed-effects model and Tukey’s post-hoc. (*p<0.05, ***p<0,001, ****p<0.0001). Control-offspring: blue circles, HTX-offspring: red squares, and HTX+T_4_-offspring: green triangles.

Subsequently, two specific tests were employed to evaluate two different dimensions of social behavior. The first test was tube dominance, design to assess socially aggressive behavior, aiding in the differentiation between subordinate and dominant statuses based on the principles of the hierarchy species theory (61). The test involves positioning two mice from distinct experimental groups at opposite entrances of a transparent hollow tube and allowing them to enter. Once they meet in the middle, a confrontation occurs, with one of the two compelling its opponent to retreat to exit. To evaluate the aggressive behavior of the HTX-gestated offspring, the following confrontation design was employed: Control-offspring vs. HTX-offspring, HTX+T_4_-offspring vs. HTX-offspring, and Control-offspring vs. HTX+T_4_-offspring (details in the Materials and Methods section). The percentage of wins for each experimental group was determined and provided in **Figure 4** and **Supplementary Table 1-1.3**. The findings revealed that both female and male HTX-offspring displayed lower winning percentages when confronted with their respective female and male Control-offspring (**Figure 4A**, **Supplementary Table 1-1.3a**), and HTX+T_4_-offspring (**Figure 4B**, **Supplementary Table 1-1.3b**), suggesting that the HTX-gestated offspring have a subordinate status in the context of confrontation. Additionally, when Control-offspring was confronted with HTX+T_4_-offspring, regardless the sex, no significant differences in the winning percentage were detected (**Figure 4C**, **Supplementary Table 1-1.3c**).

**Figure 5:**
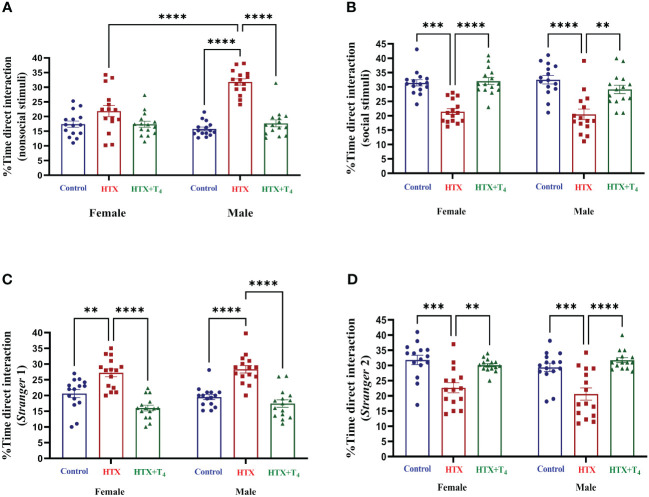
Female and male HTX-offspring display decreased direct social interaction preference compared to Control-offspring and HTX+T_4_-offspring. Offspring from the three experimental groups were subjected to analyze their social interaction abilities with the three-chamber social preference test on P64 (see Materials and methods). In the “Stranger 1” step, mice were exposed to a nonsocial and a social stimulus. **(A)** Percentage of time of direct interaction with the nonsocial stimulus and **(B)** Percentage of time of direct interaction with the social stimulus. In the “Stranger 2” step, mice were exposed to interact with the social stimulus from the first step but now renamed as “Stranger 1” step and the nonsocial stimuli was replaced with a new social incentive named “Stranger 2”. **(C)** Percentage of time of direct interaction with the S1 stimulus and **(D)** Percentage of time of direct interaction with the S2 stimulus. N = 15 per group and sex. Data are presented as mean ± S.E.M. Multiple comparisons between experimental groups and sexes were analyzed by Mixed-effects model and Tukey’s post-hoc. (**p<0.01, ***p<0,001, ****p< 0.0001). Control-offspring: blue circles, HTX-offspring: red squares, and HTX+T_4_-offspring: green triangles.

The second social aspect evaluated was the social interaction preferences through the three-chamber social preference test (62). Mice from the three experimental groups were exposed to interact with different stimuli placed in opposite compartments of a three-chamber device to assess social preferences (details provided in Materials and methods). The percentage of time spent in each compartment and the number of entrances are illustrated in **Supplementary Table 1-1.4**, **Supplementary Figures 1**, **2**. The percentage of time spent for direct interaction with each stimulus is shown in **Figure 5** and **Supplementary Table 1-1.4**. During the first step of the test, female HTX-offspring spent more time in the nonsocial compartment only compared to the HTX+T_4_ offspring, while male HTX-offspring spent more time in the nonsocial compartment compared to both the Control and HTX+T_4_ offspring (**Supplementary Table 1-1.4a**, **Supplementary Figure 1A**). Likewise, male HTX-offspring spent more time in the nonsocial compartment even compared to female HTX-offspring (**Supplementary Table 1-1.4a**, **Supplementary Figure 1A**). Additionally, only male HTX-offspring made a higher number of entrances to the nonsocial compartment (**Supplementary Table 1-1.4a**, **Supplementary Figure 1B**) and spent more time directly interacting with the nonsocial stimulus compared to both the Control and HTX+T_4_ offspring and even compared to female HTX-offspring (**Figure 5A**, **Supplementary Table 1.4c**). Conversely, only male HTX-offspring spent less time in the social compartment (**Supplementary Table 1-1.4d**, **Supplementary Figure 1C**), however, the number of entrances into the social compartment remained similar between experimental groups and sexes (**Supplementary Table 1-1.4e**, **Supplementary Figure 1E**). Finally, both female and male HTX-offspring spent less time directly interacting with the social stimulus compared to the Control and HTX+T_4_ offspring (**Figure 5B**, **Supplementary Table 1-1.4f**).

In the second phase of the test, the nonsocial stimulus was replaced with a new stranger mouse (referred to as the ‘*de novo* stimulus’ or ‘Stranger 2’), while maintaining the first stranger mouse, now renamed as ‘Stranger 1’. Despite no differences were identified in the time spent in the Stranger 1 compartment (**Supplementary Table 1-1.4g**, **Supplementary Figure 2A**), only male HTX-offspring made more entries to that compartment compared to Control and HTX+T_4_ offspring (**Supplementary Table 1-1.4h**, **Supplementary Figure 2B**). Additionally, both female and male HTX-offspring spent more time directly interacting with the Stranger 1 stimulus compared to Control and HTX+T_4_ offspring (**Figure 5C**, **Supplementary Table 1-1.4i**). Conversely, HTX-offspring (both sexes) spent less time in the Stranger 2 compartment (**Supplementary Table 1-1.4j**, **Supplementary Figure 3C**), but only females displayed a lower number of entrances to the Stranger 2 compartment (**Supplementary Table 1-1.4k**, **Supplementary Figure 3D**). Finally, both female and male HTX-offspring spent less time directly interacting with the *de novo* stimulus compared to Control and HTX+T_4_ offspring (**Figure 5D**, **Supplementary Table 1-1.4l**). Complementing these results with the first step, the HTX-gestated offspring show a reduced preference for interacting with the social stimuli but exhibit a stronger preference for interacting with the same social stimuli when the object is replaced with a second unfamiliar one in the second step of the test. These findings reinforce the notion of impaired interaction abilities resembling ASD-like behavior in this offspring.

### 3.3 Gestational HTX elevates proinflammatory markers in both female and male offspring

Beyond behavioral alterations, immune dysregulation has also been associated with ASD, mainly by the determination of proinflammatory cytokines in blood samples of patients and the examination of neuroinflammatory traits and immune cell populations in secondary lymphoid organs in mouse models (45, 46). Hence, we investigated whether gestational HTX leads to ASD-like inflammatory status in offspring.

**Figure 6:**
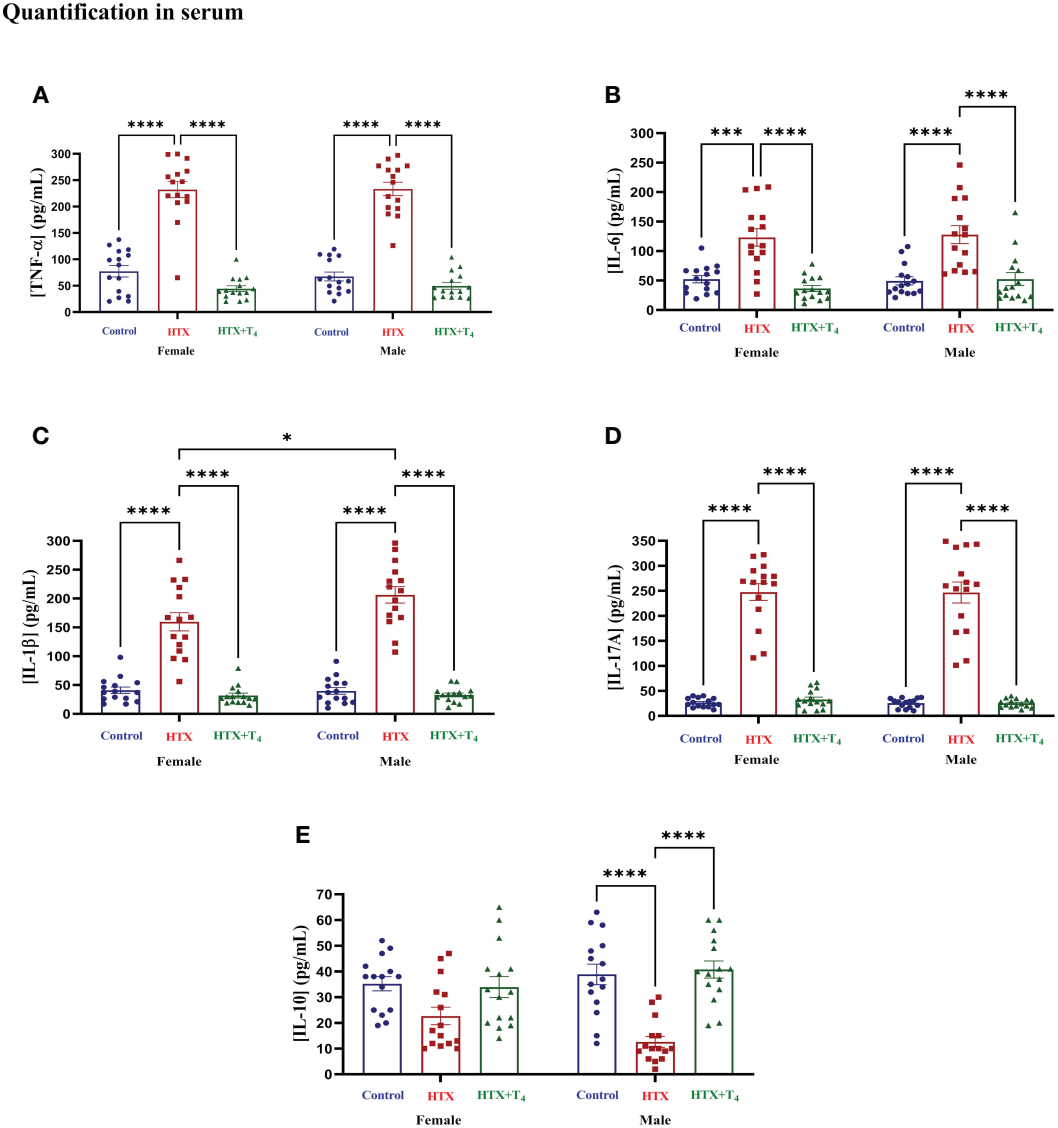
Female and male HTX-offspring has an increased concentration of pro-inflammatory cytokines in blood, while only male showed a reduced concentration of IL-10. Offspring from the three experimental groups were euthanized on P65, and the blood was extracted by cardiac puncture. The serum was isolated and used as samples (100 μL each) for the following cytokines determination by using sandwich ELISA: **(A)** TNF-α, **(B)** IL-6, **(C)** IL-1β, **(D)** IL-17A, and **(E)** IL- 10. N = 15 per group and sex. Data are presented as mean ± S.E.M. Multiple comparisons between experimental groups and both sexes were analyzed by Mixed-effects model and Tukey’s post-hoc. (*p<0.05, ***p<0,001, ****p<0.0001). Control-offspring: blue circles, HTX-offspring: red squares, and HTX+T_4_-offspring: green triangles.

**Figure 7:**
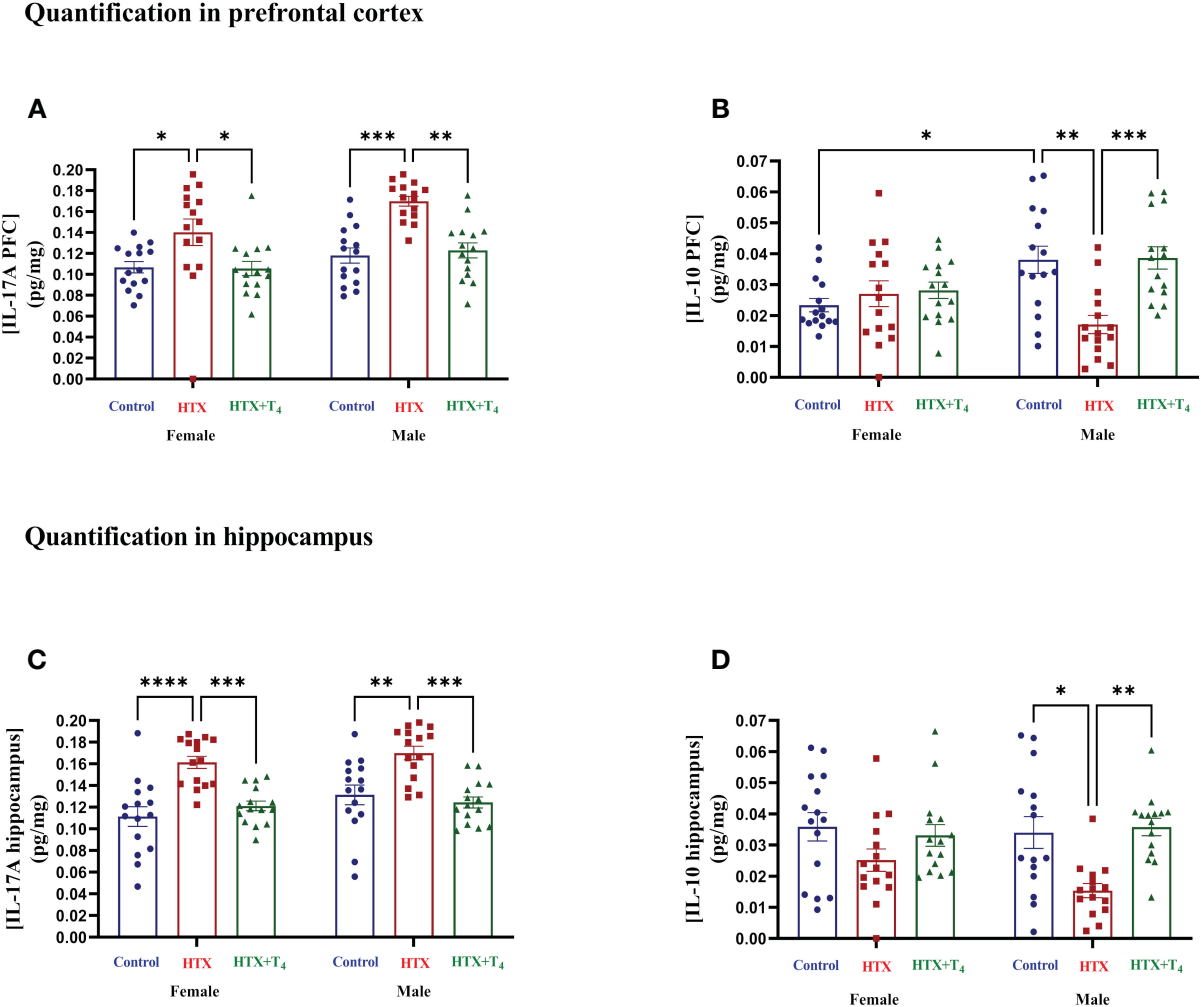
The HTX-gestated offspring of both sexes has higher levels of IL-17A in the prefrontal cortex and hippocampus than the control groups and reduced IL-10 only in males. Progenies of the three experimental groups were euthanized on P65, and PFC and hippocampus were isolated. Total proteins were extracted from these tissues and 0.5 mg per sample were used for the measurement of IL-17A and IL-10 by sandwich ELISA. **(A)** IL-17A in the PFC, **(B)** IL-10 in the PFC, **(C)** IL-17A in the hippocampus, and **(D)** IL-10 in the hippocampus. Data was normalized to mg of proteins. N = 15 per experimental group and sex. Data are presented as mean ± S.E.M. Multiple comparisons between experimental groups and both sexes were analyzed by Mixed-effects model and Tukey’s post-hoc. (*p<0.05, **p<0.01, ***p<0,001, ****p<0.0001). Control-offspring: blue circles, HTX-offspring: red squares, and HTX+T_4_-offspring: green triangles.

After conducting the behavioral tests, mice were euthanized, and specific cytokines were measured from serum samples (refer to Materials and Methods) (**Figure 6**, **Supplementary Table 2-2.1**). The results indicate that both female and male HTX-offspring had increased concentrations of the proinflammatory cytokines TNF-α (**Figure 6A**, **Supplementary Table 2-2.1a**), IL-6 (**Figure 6B**, **Supplementary Table 2-4.1b**), IL-1β (**Figure 6C**, **Supplementary Table 2-2.1c**), and IL-17A (**Figure 6D**, **Supplementary Table 2-2.1d**), in comparison to Control and HTX+T_4_ offspring. Additionally, IL-1β was even higher in the male HTX-offspring than in females (**Figure 6C**, **Supplementary Table 2-2.1c**). The anti-inflammatory cytokine IL-10 was significantly reduced only in male HTX-offspring compared to male Control and HTX+T_4_ offspring (**Figure 6E**, **Supplementary Table 2-2.1e**). The increased serum concentration of proinflammatory cytokines in the HTX-offspring is in concordance with ASD-like molecular features. Considering the neuroinflammatory component of ASD (47), IL-17A and its counterpart IL-10 were quantified in brain regions related to social cognition (prefrontal cortex (PFC) and hippocampus) (**Figure 7**, **Supplementary Table 2-2.2**). In the PFC, both female and male HTX-offspring had an increased concentration of IL-17A (**Figure 7A**, **Supplementary Table 2-2.2a**), while a reduction of IL-10 in the PFC was only observed in male HTX-offspring, compared to Control and HTX+T_4_ offspring (**Figure 7B**, **Supplementary Table 2-2.2b**). Interestingly, the male Control-offspring had a higher content of IL-10 in the PFC compared to female Control-offspring (**Figure 7B**, **Supplementary Table 2-2.2b**). Moreover, IL-17A in the hippocampus was increased in HTX-offspring of both sexes (**Figure 7C**, **Supplementary Table 2-2.2c**), whereas IL-10 was reduced only in the hippocampus of male HTX-offspring compared to Control and HTX+T_4_ offspring (**Figure 7D**, **Supplementary Table 2-2.2d**). These results suggest that the HTX-offspring exhibits a sex-dependent neuroinflammatory-like component which can also be indicative of an ASD-like phenotype.

**Figure 8:**
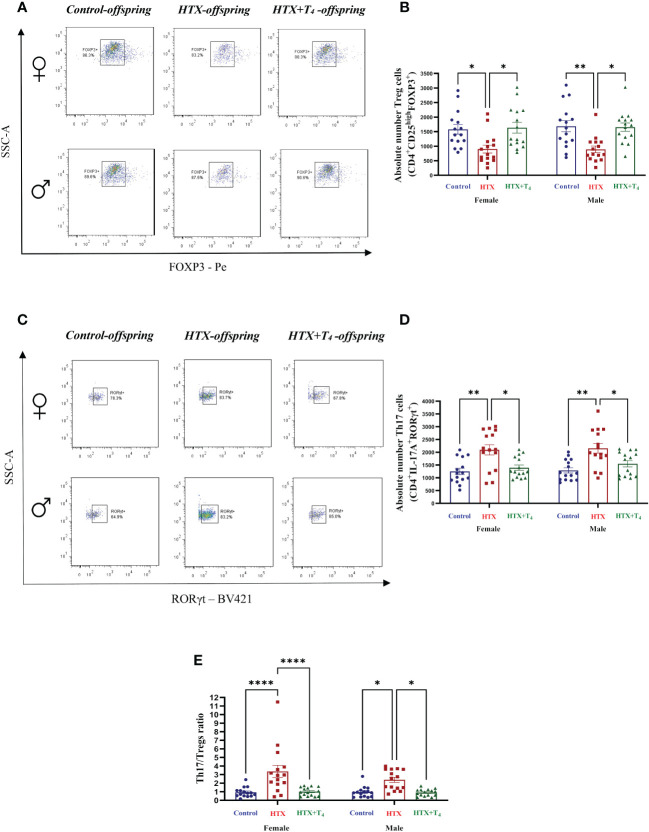
The HTX-offspring of both sexes has an imbalance in the Th17/Tregs ratio inclined towards increased Th17 cell number. Progenies from the three experimental groups were euthanized on P65, and spleens were removed. Total splenocytes were purified and a portion of these cells was *in vitro* stimulated using PMA-Ionomycin-BFA (see Materials and methods). The population of Tregs and Th17 were quantified by flow cytometry. **(A)** Representative dot plots of FOXP3-positive cells, **(B)** The graph shows the absolute number of FOXP3-positive cells, **(C)** Representative dot plots of RORγt-positive cells, **(D)** the graph shows the absolute number of RORγt-positive cells, and **(E)** the graph shows the Th17/Tregs ratio. N = 15 per group and sex. Data are presented as mean ± S.E.M. Multiple comparisons between groups and both sexes were analyzed by Mixed-effects model and Tukey’s post-hoc. (*p<0.05, **p<0.01, ****p<0,0001). Control-offspring: blue circles, HTX-offspring: red squares, and HTX+T_4_-offspring: green triangles.

**Figure 9:**
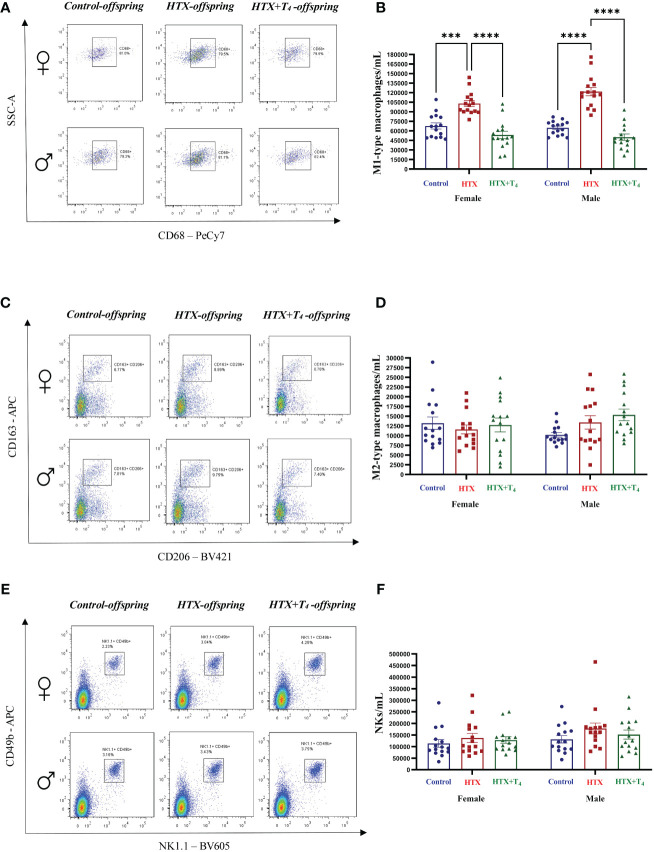
M1-type macrophages are increased in HTX-offspring’s spleen of both sexes, while M2-type macrophages and NK cells remain similar among experimental groups. Splenic innate immune cells were quantified by flow cytometry (see Materials and Methods). **(A)** Representative dot-plots of CD68-positive cells, **(B)** The graph shows the absolute number of CD68-positive cells, **(C)** Representative dot-plots of CD206/CD163 double-positive cells, **(D)** the graph shows the absolute number of CD206/CD163 double-positive cells, **(E)** Representative dot-plots of NK1.1/CD49b double-positive cells, and **(F)** the graph shows the absolute number of NK1.1/CD49b double-positive cells. N = 15 per group and sex. Data are presented as mean ± S.E.M. Multiple comparisons between experimental groups and both sexes were analyzed by Mixed-effects model and Tukey’s post-test. (***p<0.001, ****p<0.0001). Control-offspring: blue circles, HTX-offspring: red squares, and HTX+T_4_-offspring: green triangles.

Finally, to broaden our comprehension of the immunological aspect of the HTX-offspring regarding the proportion of immune cell populations (48, 49), total splenocytes were isolated, and myeloid and specific T cells were assessed by flow cytometry. Total splenocytes were subjected to an *in vitro* stimulation with PMA, ionomycin, and BFA. Thereafter, harvested cells were labeled with appropriate antibodies to identify Tregs and Th17 cells and analyzed by flow cytometry (see Materials and methods). The results are presented in **Figure 8** and the employed gating strategy for Treg and Th17 cell selection is shown in **Supplementary Figures 3A** and **B**, respectively. The findings revealed that both female and male HTX-offspring had a lower absolute number of CD4^+^CD25^high^FOXP3^+^ cells (Tregs), compared to Control and HTX+T_4_ offspring (**Figures 8A, B**). Conversely, an increased absolute number of CD4^+^IL-17A^+^RORγt^+^ cells (Th17) was observed in HTX-offspring of both sexes, also compared to Control and HTX+T_4_ offspring (**Figures 8C, D**). Consistent with these findings, both female and male HTX-offspring had a higher Th17/Treg ratio (**Figure 8E**). Myeloid cells, particularly M1/M2-like macrophages and NK cells were also evaluated in splenocytes by flow cytometry. The results are depicted in **Figure 9** and the employed gating strategy is shown in **Supplementary Figures 4A-C**. Similarly, both female and male HTX-offspring had a higher absolute number of CD45^+^CD3^-^F4/80^+^CD80^+^CD68^+^ cells (M1-like macrophages), than the Control and HTX+T_4_ offspring (**Figures 9A, B**). Nonetheless, no differences were observed in the absolute numbers of CD45^+^CD3^-^F4/80^+^CD206^+^CD163^+^ cells (M2-like macrophages) (**Figures 9C, D**) and CD45^+^CD3^-^CD11b^+^NK1.1^+^CD49b^+^ cells (NKs) (**Figures 9E, F**). These findings, in conjunction with the previous ones, indicate that the HTX-offspring also exhibits an imbalanced proportion of immune cells, overall pointing to an exacerbated proinflammatory status that aligns with an ASD-like phenotype.

### 3.4 Gestational HTX increases the expression of hippocampal NLGN3 and HOMER1 in male offspring

**Figure 10:**
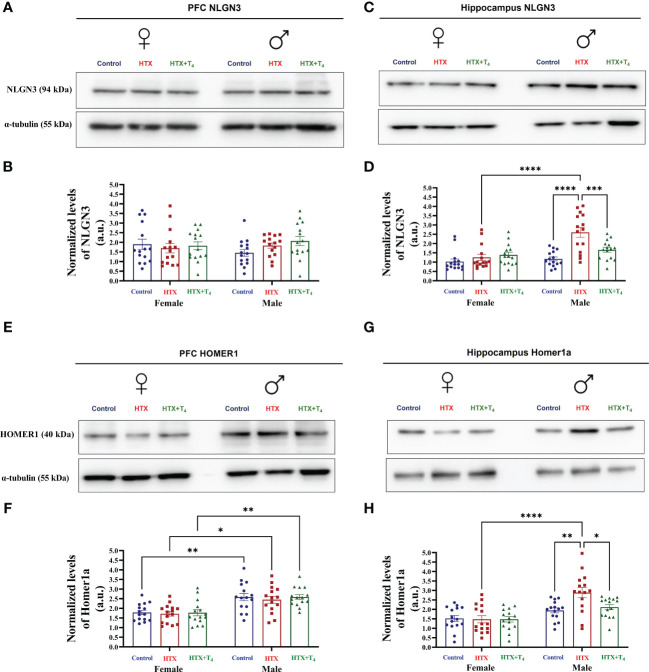
The expression of hippocampal NLGN3 and HOMER1 is increased in male HTX-offspring. Progenies from the three experimental groups were euthanized on P65, and prefrontal cortex (PFC) and hippocampus were isolated. Total proteins were extracted from these tissues and the relative expression of Neuroligin 3 (NLGN3) and HOMER1 from the PFC and hippocampus were evaluated by western blot in each experimental group. **(A)** Representative photography of the western blot of NLGN3 from the PFC, **(B)** the graph shows the normalized relative expression of NLGN3 in the PFC, **(C)** representative photography of the western blot of NLGN3 from the hippocampus, **(D)** the graph shows the normalized relative expression of NLGN3 from the hippocampus, **(E)** representative photography of the western blot of HOMER1 from the PFC, **(F)** the graph shows the normalized relative expression of HOMER1 in the PFC, **(G)** representative photography of the western blot of HOMER1 from the hippocampus, and **(H)** the graph shows the normalized relative expression of HOMER1 from the hippocampus. N =15 per group and sex. a.u means arbitrary units. Data are presented as mean ± S.E.M. Multiple comparisons between experimental groups were analyzed by Mixed-effects model and Tukey’s post-hoc. (*p<0.05, **p<0.01, ***p<0.001, ****p<0.0001). Control-offspring: blue circles, HTX-offspring: red squares, and HTX+T^4^-offspring: green triangles.

The effects of gestational HTX on offspring behavior and immune status encourage exploration into whether this condition also affects the expression of glutamatergic proteins, such as NLGN3 and HOMER1, whose altered levels are associated with ASD-like behaviors in mice (72). For this purpose, total proteins from both the PFC and hippocampus were isolated, and the expression of NLGN3 and HOMER1 was determined by using western blot (refer to Material and methods for details). The results of this analysis are shown in **Figure 10**.

The expression levels of NLGN3 in the PFC were comparable among the HTX, Control, and HTX+T_4_ offspring, regardless the sex (**Figures 10A, B**), however, only male HTX-offspring showed a significant increase in the expression levels of hippocampal NLGN3 compared to male Control and HTX+T_4_ offspring (**Figures 10C, D**). At the same time, the expression levels of hippocampal NLGN3 in male HTX-offspring was significantly higher than in female HTX-offspring (**Figures 10C, D**). On the other hand, the expression levels of HOMER1 in the PFC was similar when comparing the HTX, Control, and HTX+T_4_ offspring of the same sex (**Figures 10E, F**), however, it is worth noting that the expression levels of HOMER1 in the PFC of males in the Control, HTX, and HTX+T_4_ offspring was simultaneously higher than in females of the Control, HTX, and HTX+T_4_ offspring (**Figures 10E, F**). Finally, only male HTX-offspring showed higher expression levels of hippocampal HOMER1 compared to male Control, HTX+T_4_, and even female HTX-offspring (**Figures 10G, H**). These results indicate that the effects of gestational HTX on the offspring go beyond behavior and immune response, but also induce sex-dependent alterations in the expression of glutamatergic proteins, which collectively can be interpreted as ASD-like outcomes.

Additionally, the results of this work were also collectively analyzed (without separation by sex) and are presented from **Supplementary Figures 5**–**14**.

### 3.5 Maternal T_4_ levels are correlated with specific behavioral and proinflammatory outcomes of the offspring

In this study, gestational HTX was defined as a reduction of maternal tT_4_ within a range of [0.1 – 1.9] (μg/dL), whereas the Control and HTX+T_4_ ranges were established at [2.2 – 4.5] (μg/dL). Nonetheless, it is relevant to note that these ranges were defined within the context of natural variability. For this reason, to investigate whether maternal T_4_ levels could influence the development of ASD-like phenotypes in the offspring, a Pearson correlation analysis was conducted (73) to measure the association between maternal T_4_ levels (considering all HTX, Control, and HTX+T_4_ dams’ levels) and all the observed outcomes (**Table 1**, **Supplementary Figure 15**).

**Table 1:** Maternal T_4_ is correlated with ASD-like outcomes of the offspring.

Parameter	ASD-like outcomes	*r*	*p* value
Maternal T_4_ levels	%Buried Marbles	0,578	(****) *p*<0.0001
	N° entries to open arms	0,591	
	Direct interaction with Social stimuli	0,559	
	IL-1β in serum	-0,641	
	IL-17A in serum	-0,721	
	IL-10 in serum	0,578	

Pearson correlations were employed to associate maternal T_4_ with all measured outcomes in the offspring of both sexes. The table displays correlations specifically within the range of 0.5 to 1.0, representing “medium to high” associations.

The results revealed significant positive correlations between maternal T_4_ levels and the percentage of buried marbles (*r*=0.578, *p*<0.00001), the number of entries into open arms during the EPM test (*r*=0.591, *p*<0.00001), the percentage of time directly interacting with social stimuli in the Three-chamber test (*r*=0.559, *p*<0.00001), and IL-10 concentration in serum (*r*=0.578, *p*<0.00001). Conversely, significant inverse associations were noted between maternal T_4_ levels and IL-1β (*r*=-0.641, *p*<0.00001) and IL-17A (*r*=-0.721, *p*<0.00001) levels in the blood (**Table 1**). These associations suggests that maternal thyroid status influence the development of ASD-like phenotypes in the offspring.

Additional significant correlations between behavioral parameters and proinflammatory outcomes are shown in **Supplementary Figures 17A, B**. Interestingly, the IL-1β levels, regardless of sex, were negatively correlated with specific behavioral parameters, including the percentage of buried marbles in the MB test (*r*=-0.580, *p*<0.00001), the number of entries into the open arms in the EPM test (*r*=-0.600, *p*<0.00001), the time of direct interaction with the social stimuli in the three-chamber test (*r*=-0.586, *p*<0.00001), and positively correlated with the time of direct interaction with the Stranger 1 stimuli in the three-chamber test (*r*=0.653, *p*<0.00001) (**Supplementary Figure 15**). At the same time, the IL-1β levels were positively correlated with other proinflammatory outcomes, including the blood levels of IL-17A (*r*=0.632, *p*<0.00001) and TNF-α (*r*=0.649, *p*<0.00001), and the number of M1-like macrophages in the spleen (*r*=0.588, *p*<0.00001) (**Supplementary Figure 15**). Similar trends are evident in the associations between the blood levels of IL-6, IL-17A, and TNF-α with behavioral parameters (**Supplementary Figure 15**). Moreover, the levels of IL-17A in the hippocampus were negatively correlated with the percentage of buried marbles (*r*=-0.640, *p*<0.00001), while the IL-17A levels in the PFC were positively correlated with the levels of TNF-α in blood (*r*=0.634, *p*<0.00001) (**Supplementary Figure 15**). No significant correlations were identified regarding the levels of IL-10 in either the PFC or hippocampus (**Supplementary Figure 15**). At the cellular level, the Th17/Tregs ratio was negatively correlated with the MB test performance (*r*=-0.603, *p*<0.00001), while the number of M1-like macrophages showed a negative correlation with the number of entries to the exposed arms in the EPM test (*r*=-0.557, *p*<0.00001), and a positive correlation with the direct interaction time with the Stranger 1 stimuli (*r*=0.666, *p*<0.00001), and the blood levels of IL-1β (*r*=0.588, *p*<0.00001), IL-6 (*r*=0.582, *p*<0.00001), IL-17 (*r*=0.636, *p*<0.00001), and TNF-α (*r*=0.630, *p*<0.00001) (**Supplementary Figure 15**).

## 4 Discussion

In this work, we present evidence indicating that gestational HTX induces long-term effects in offspring of both sexes. These effects include ASD-like alterations in behavior, elevated proinflammatory markers in blood, spleen, prefrontal cortex, and hippocampus, as well as sex-dependent alterations in the expression of glutamatergic proteins NLGN3 and HOMER1 in the hippocampus.

In light of previous evidence from associative studies in humans linking gestational HTX with ASD traits in offspring (17, 18), a variety of behavioral tests were conducted to evaluate ASD-like behaviors in mice gestated with HTX, with the intention of validating such association, and considering both females and males to explore potential sex-specific effects (**Figures 2** **-** **5**). The reason for utilizing at least four tests is that the ASD-like behaviors may not consistently manifest across all tests. This variability is evident from behavioral assessments conducted on genetically modified mouse models associated with ASD-like conditions, such as *Ube3a*, *Pten*, *Nlgn3*, *Shank3*, *Mecp2*, and *Fmr1* (74, 75). Additionally, chemically induced ASD-like models during gestation, including the use of neuropsychiatric drugs like valproic acid (VPA) and molecules mimicking infections such as lipopolysaccharide (LPS) and polyinosinic:polycytidylic acid (poly(I:C)), have also exhibited variability in the expression of ASD-like behaviors (74, 75).

In the marble burying test, it is expected that mice exhibiting repetitive behavior bury an increased number of marbles than control mice (57, 58). Nonetheless in this case, both female and male HTX-offspring significantly buried fewer glass marbles compared to Control and HTX+T_4_ offspring (**Figure 2**, **Supplementary Table 1-1.1**), suggesting that the HTX-offspring does not exhibit repetitive behavior. In contrast, it has been reported that mouse offspring exposed to other *in utero* alterations, including maternal inflammation (MIA-offspring) and the exposition to teratogens such as VPA (VPA-offspring), bury more marbles than the control in the MB test, inferring that these mice display repetitive behavior (76–78). Despite this result, it is reported that the behavior of the HTX-offspring in the MB test can be associated with impaired hippocampal function in terms of impaired synaptic transmission (57, 58). This finding further supports previous research conducted on the HTX-gestated offspring, which showed deficient long-term potentiation (LTP) induction and compromised spatial learning and memory functions (54). A widespread comorbid condition in ASD is anxiety (71), prompting the evaluation of anxious-like behavior in the HTX-offspring using the EPM test. The HTX-offspring displayed a significant preference for closed spaces over exposed arms (**Figure 3**, **Supplementary Tables 1-1.2a-d**), mirroring observations in MIA and VPA offspring (79, 80). These findings suggest a shared pattern of anxiety-related responses among HTX, MIA, and VPA offspring, which are associated with ASD-like manifestations in mice. This resemblance underscores the high prevalence of anxiety as a primary comorbid condition in ASD (71, 79, 80). On the other hand, traits associated with social hierarchy, including aggressive and submissive behaviors, are widely addressed in neurodevelopmental disorders research (61). To assess these behaviors, the tube dominance test was conducted, revealing that HTX-offspring displayed subordinate behavior in comparison to Control and HTX+T_4_ offspring (**Figure 4**, **Supplementary Tables 1-1.3a-c**). The behavioral patterns exhibited by HTX-offspring in this regard bore resemblance to those observed in genetically modified mouse models of ASD, such as SHANK2 and SHANK3 mutant mice (81). These convergences in behavior raise the possibility that HTX exposure may affect the expression of genes related to prenatal ASD development, potentially affecting the normal neurodevelopment (81). Finally, the three-chamber social preference test was conducted to assess social interaction preferences, crucial in ASD diagnosis (62). The male HTX-offspring exhibited a significantly increased preference for inanimate objects over social stimuli, maintaining this lack of interest until the introduction of a novel social stimulus (**Figure 5**, **Supplementary Tables 1-1.4**a-l). While the females also interacted for less time with the social stimulus, they did not show greater interest in the nonsocial stimulus, suggesting overall sex-dependent ASD-like behavioral manifestations in the HTX-offspring. Notably, the HTX-offspring, regardless of sex, showed extended interaction with the first social stimulus compared to the new one (**Figure 5**, **Supplementary Tables 1-1.4a-l**). These trends were concordant with previous observations in different ASD-like models, including MIA and VPA offspring, where males tend to manifest more pronounced deficits in social interaction abilities (82, 83). In the context of the three-chamber test, these results are indicators of impaired social skills, which are widely recognized to correlate with recurring ASD-like behaviors in both humans and mice (84–86).

The behavioral manifestations observed in the HTX-offspring can be associated with ASD-like features, thus validating previous retrospective studies in humans. However, it’s important to note that the conclusions drawn from this work regarding behavior are limited to the tests described here. Hence, this study opens the possibility of conducting a more exhaustive behavioral analysis to evaluate other ASD-like behavioral facets comprehensively.

Transitioning into the molecular analyses, ASD has an immune component mainly featured by increased concentration of proinflammatory cytokines in blood samples of patients (45, 46). Female and male HTX-offspring displayed elevated levels of IL-6, IL-17A, IL-1β, and TNF-α in their blood compared to Control and HTX+T_4_ offspring (**Figures 6A-D**, **Supplementary Tables 2-2.1a-d**). Conversely, there was a decrease in the levels of the anti-inflammatory cytokine IL-10, but this effect was specific to male HTX-offspring (**Figure 6E**, **Supplementary Table 2-2.1e**). These findings align with research reported in children diagnosed with ASD (45, 46), suggesting that this alteration may potentially serve as a hallmark of ASD disorders, considering its significant prevalence among diagnosed individuals (87–89). Interestingly, at the molecular level, IFN-γ and IL-1β have recently been discovered to stimulate GABAergic neurons, resulting in hyper-connectivity in fronto-cortical brain regions and subsequent impairments in social and anxious behaviors (90). Furthermore, the same study reports a strong statistical correlation between increased levels of IFN-γ and IL-1β and alterations in social behaviors not only in mice but also in fish and flies (90). The proinflammatory status observed in the HTX-offspring leads us to speculate that the elevated proinflammatory cytokines may affect neuronal functions, potentially translating into the observed ASD-like behavioral alterations in sociability and anxiety. With this in mind, we also infer that the increased concentration of IL-1β in the blood of male HTX-offspring compared to female HTX-offspring might be associated with the heightened interest of males in direct interact with the nonsocial stimuli in the three-chamber social test (**Figure 5A**). The rationale behind these associations is justified by the fact that as the HTX-offspring exhibit an elevated concentration of IL-1β in the blood (**Figure 6C**), there is a corresponding decrease in the number of buried marbles, the number of entrances to the open arms, the time of direct interaction with the nonsocial stimuli, and more time interacting with the Stranger 1 stimuli (**Supplementary Figure 15**). These alterations further support the notion that heightened levels of proinflammatory cytokines in the HTX-offspring could impact their behavior and potentiate their proinflammatory status.

Neuroinflammation, triggered by the local increase in brain regions of IL-17A, is a significant feature in ASD pathophysiology (91). The levels of IL-17A in both the PFC and hippocampus of HTX-offspring were notably higher compared to those in the Control and HTX+T_4_ offspring of both sexes (**Figures 7A, C**, **Supplementary Tables 2-2.2a, c**). This observation is consistent with previous research showing that MIA-offspring exhibited increased proinflammatory cytokines like IL-17A, TNF-α, IL-1β, and IL-6, accompanied by synaptic deficiencies in the PFC and increased Iba1 mark in astrocytes of brain cortex and cerebellum (92–95). Similar trends have been observed in the VPA-offspring, where also microglial activation and structural changes were apparent in specific brain regions like the PFC and hippocampus (96). Interestingly, a previous study demonstrated that microglia derived from the HTX-gestated offspring exhibited heightened reactivity compared to those from the offspring gestated under euthyroid conditions when exposed to a proinflammatory stimulus (52). This finding reinforces the notion of the presence of neuroinflammatory-like traits in HTX-offspring, indicating a greater propensity to respond more severely to inflammation (52). We also identified a reduction in IL-10, but only within the PFC and hippocampus of male HTX-offspring (**Figures 7B, D**, **Supplementary Tables 2-2.2b, d**). This discovery is consistent with previously reported sex-specific patterns of neuroinflammation, which may potentially contribute to the increased susceptibility of males to neurodevelopmental disorders (97–99). It has been demonstrated that elevated levels of IL-17A exerts detrimental effects on CNS function (100). Specifically, neuroinflammation triggers the activation of T-cells, macrophages, microglia, and astrocytes in the brain, resulting in decreased hippocampal neurogenesis through apoptotic pathways, heightened GABAergic signaling, and upregulation of MHC class I and II protein complexes (91, 100, 101). Additionally, IL-17A induces reduced dendritic spine density and enhances reactive microglial phenotypes (91, 100, 101). These alterations in the central nervous system (CNS) have been correlated with ASD-like behavioral outcomes, particularly affecting social interactions (91, 100, 101). Hence, we infer that the increased levels of IL-17A in brain regions related to social cognition of the HTX-gestated offspring (**Figure 7**, **Supplementary Figure 15**) may further contribute to the cognitive impairments evidenced by the performance of these offspring in the marble burying test (**Figures 2**, **Supplementary Figure 15**).

Alterations in immune cell populations have been observed in ASD-like models (45, 46, 48). The differences observed on immune cell populations demonstrate an inherent imbalance between tolerogenic and inflammatory T cells in female and male HTX-offspring (**Figures 8A-E**). This imbalance results from an augmented number of Th17 cells and a decreased Treg cell number, leading to an elevated Th17/Tregs ratio in this offspring (**Figures 8A-E**). This observation aligns with earlier reports demonstrating that autistic children exhibit a heightened proportion of Th17 cells in comparison to Treg cells (65). These findings are also consistent with prior reports indicating that adult HTX-offspring exhibit a diminished tolerogenic capacity when confronted with the challenge of experimental autoimmune encephalomyelitis (EAE) (53, 102). Complementary, their naïve T cells exhibited a reduced inclination to differentiate into the Treg cell phenotype which could be a possible contribution to the diminished Treg cell number observed (53). Additionally, we detected an increase in the absolute number of M1-like macrophages in HTX-offspring, while the absolute count of M2-like macrophages and NK cells remained consistent across experimental groups and both sexes (**Figures 9A-D**). Given that M1-like macrophages exhibit a pro-inflammatory profile (103), this imbalance agrees with the notion of an underlying basal inflammatory state in HTX-offspring.

The results concerning the proinflammatory status of HTX-gestated offspring suggest that elevated levels of specific cytokines may impair neuronal functions in relevant brain regions, thereby contributing to the induction of behavioral and cognitive alterations resembling ASD-like manifestations. Additionally, the proinflammatory state of HTX-offspring appears to reinforce itself, as evidenced by the correlations found between cytokine levels and the mentioned immune cell populations.

Disruptions in glutamatergic protein expression represent another relevant feature associated with ASD disorders (104, 105). In fact, autism can be conceptualized as a synaptic disorder characterized by an equilibrium disruption between excitatory and inhibitory synapses (106). Focusing on the glutamatergic synapses, alterations in scaffolding proteins expression have been implicated in the manifestation of autistic traits (107–109). In this work, we identified an augmented expression of NLGN3 and HOMER1 primarily in the hippocampus of male HTX-offspring (**Figures 10D, H**). It has been documented that an elevated HOMER1 expression has been linked to ASD-like behaviors as well as deficits in fear conditioning (42). In addition, the expression patterns of these proteins were also region-specific within the brain. For instance, the PFC did not display substantial changes in the expression of NLGN3 or HOMER1 across experimental groups (**Figures 10B, F**). However, a basal elevation in HOMER1 levels was noted in males across all three experimental groups when compared to females (**Figure 10F**). The mechanisms underlying the sex-dependent expression of these proteins remain elusive. Nonetheless, these intriguing observations could potentially offer valuable insights into unraveling the molecular foundations contributing to the heightened susceptibility of males in developing an ASD-like condition compared to females. Furthermore, it is proposed that neuroinflammation may influence the expression of glutamatergic proteins (107–109). However, no correlations were found between the levels of NLGN3 or HOMER1 expression and IL-17 or IL-10 in the PFC or hippocampus, nor with the behavioral parameters assessed in this study (**Supplementary Figure 15**). This suggests that alterations in hippocampal HOMER1 expression in males are not influenced by IL-17A levels, nor vice versa, and that these alterations are not directly associated with the observed behavioral changes. Nonetheless, further investigation is warranted to elucidate the complex interplay between neuroinflammation and glutamatergic protein expression in the context of ASD-like manifestations in the HTX-gestated offspring.

At the same time that we performed the data analyses by separating them according to sex with the intention of exploring sex-dependent outcomes, we conducted the analyses by combining the female and male data as a single Control, HTX, and HTX+T_4_ experimental group (**Supplementary Figures 5**–**14**). In general, we observed similar results when combining the data. However, in terms of the duration of direct interaction with Stranger 1 stimuli (**Supplementary Figure 9C**), TNF-α blood levels (**Supplementary Figure 10A**), and the absolute number of M1-like macrophages in the spleen (**Supplementary Figure 15A**), the levels of each parameter in the HTX+T_4_-offspring were statistically lower than those in the HTX-offspring and even the Control-offspring. Another notable difference was found in the expression levels of hippocampal NLGN3 in the HTX+T_4_-offspring, which were not statistically smaller than those in the HTX-offspring as observed in the Control-offspring (**Supplementary Figure 14B**). We attribute these differences to the plausible synergistic effects of T_4_ supplementation in mitigating specific behavioral alterations and inflammatory responses in HTX-gestated offspring. Nonetheless, while this suggests a potentially promising avenue for intervention strategies aimed at correcting maternal thyroid hormone deficiency during pregnancy, it also underscores the importance of determining appropriate levels of T_4_ supplementation to avoid exacerbated synergistic effects (110, 111). In this context, we emphasize that this article focuses on a condition that decreases maternal T_4_ levels and evaluates its effects on the offspring. Therefore, we did not consider treatment solely with T_4_, as elevated maternal T_4_ levels may induce transient non-autoimmune forms of hyperthyroidism characterized by an increase in T_4_ levels with almost undetectable TSH (112). Even so, gestational hyperthyroidism is more related to increased risk of preeclampsia, preterm delivery, heart failure, and thyroid storm in the mother, and to low birth weight, intrauterine growth retardation, and in the fetus (112). On the other hand, the blood levels of IL-10 are reduced in the HTX-offspring (**Supplementary Figure 10E**), but when we separate the data according to sex, we observed that this difference is due to the male HTX-offspring (**Figure 6E**). Similarly, the levels of IL-10 in the PFC of the HTX-offspring tend to be decreased compared to Control-offspring (**Supplementary Figure 11B**), nonetheless, when we separate the data according to sex, we observed that only male HTX-offspring has reduced levels of IL-10 in the PFC (**Figure 7B**). The expression levels of HOMER1 in both the prefrontal cortex (PFC) and hippocampus of HTX offspring did not show statistical differences when compared to those of the Control and HTX+T_4_ offspring (**Supplementary Figures 14C, D**). However, had we not separated the data according to sex, it would not have been possible to identify that only males exhibited an increased expression levels of HOMER1 in the PFC and hippocampus (**Figures 10E-H**). In summary, through this study, we suggest the need to analyze ASD-like outcomes ideally by separating the data according to sex, as this concise modification in data analysis can unveil interesting perspectives on ASD-like manifestations.

Since our experimental design involved evaluating ASD-like phenotypes in mice gestated under HTX, and we contemplated using mice from the different litters (mitigating the impact of the litter effect (69)), we were able to conduct a correlation analysis between maternal T_4_ levels and the ASD-like outcomes in the offspring (**Table 1**). Notably, we identified a positive association between maternal T_4_ levels and specific behavioral parameters, alongside elevated serum IL-10 levels. Conversely, an inverse relationship was observed between maternal T_4_ levels and proinflammatory cytokines in serum (**Table 1**). Moreover, our analysis revealed associations among autism-like outcomes, showing that behavioral alterations correlated with proinflammatory traits, and these proinflammatory markers were interrelated (**Supplementary Figure 15**). These findings align with previous reports indicating correlations between prenatal inducers of ASD such as maternal inflammation, VPA treatment, exposure to teratogens or contaminants, and nutritional deficiencies with ASD-like alterations in behaviors, increased proinflammatory markers, and abnormal expression of glutamatergic proteins in the offspring (113–118). Therefore, this study provides compelling insights into the potential influence of maternal T_4_ on distinct ASD-like phenotypes in the offspring.

Prenatal inducers of ASD-like phenotypes in the offspring must occur within a specific timeframe (early embryonic days (E10-E12.5 or E14)) (113–118). Regarding gestational HTX, this specific timeframe is chosen because during this period of gestation maternal T_4_ plays an active and crucial role in the proper neurodevelopment of the fetus, closely resembling early human gestation (70, 119, 120). The mechanisms underlying how gestational HTX heightens the risk of ASD in the offspring remain unknown. Curiously, maternal autoimmune disorders affecting the thyroid gland have also been identified as risk factors for ASD-like traits in the offspring (121). Specifically, offspring gestated in mothers afflicted with Hashimoto’s thyroiditis with elevated thyroid peroxidase antibody (TPO-Ab^+^) levels were found to have approximately an 80% increased probability of developing autistic traits (121). Consequently, thyroid disorders during early pregnancy may trigger proinflammatory responses, potentially leading to morphological and epigenetic changes in fetal neurodevelopment (122, 123). This could increase the likelihood of ASD-like traits in the offspring, similar to findings MIA model (122, 123). Thus, we believe that gestational HTX could induce such ASD-like traits by inducing a maternal proinflammatory response, underscoring the intricate interplay between maternal THs and inflammation during fetal programming associated with neurodevelopmental disorders.

Overall, this research represents the first validation of a causal link between gestational HTX and ASD-like phenotypes in the offspring of both sexes—a phenomenon that is mitigated by T_4_ restoration during pregnancy. Furthermore, our associative findings suggest that the proinflammatory status of the HTX-offspring may influence their behavior through alterations in neuroinflammatory responses and glutamatergic protein expression. These insights underscore the complex interplay between maternal thyroid function, immune regulation, and neurodevelopmental outcomes in the offspring. Moving forward, elucidating the underlying mechanisms linking gestational HTX, neuroinflammation, and ASD-like phenotypes holds promise for informing preventive and therapeutic strategies for individuals at risk of neurodevelopmental disorders. Further investigations into the molecular pathways involved in these processes will be crucial for advancing our understanding and developing targeted interventions to mitigate the impact of gestational thyroid dysfunction on the offspring neurodevelopment.

## 5 Conclusion

This study provides groundbreaking evidence demonstrating that gestational HTX significantly increases the likelihood of developing ASD-like phenotypes in the offspring. Behaviorally, the HTX-exposed offspring of both sexes exhibited compromised neurocognitive functions, heightened anxious-like behaviors, reduced aggressive behavior, and a pronounced preference for interacting with inanimate stimuli over other mice. These behavioral alterations were accompanied by molecular impairments, including inflammation and altered expression of glutamatergic proteins, particularly in male offspring. Furthermore, maternal T_4_ levels were found to be associated with specific behavioral and proinflammatory ASD-like outcomes in the offspring, underscoring the pivotal role of maternal thyroid hormones in neurodevelopmental disorders such as ASD. Importantly, these findings shed light on sex-dependent factors contributing to vulnerability to neurodevelopmental disorders and underscore the importance of closely monitoring thyroid function during early pregnancy.

## Data availability statement

The original contributions presented in the study are included in the article/**Supplementary Material**. Further inquiries can be directed to the corresponding author.

## Ethics statement

The animal study was approved by Animal Welfare Committee of the Facultad de Ciencias de la Vida (Universidad Andrés Bello) (Bioethics approval certificate number 012/2021). The study was conducted in accordance with the local legislation and institutional requirements.

## Author contributions

EG-M: Conceptualization, Data curation, Investigation, Methodology, Software, Writing – original draft, Writing – review & editing. MR-R: Investigation, Methodology, Software, Writing – review & editing. MO: Data curation, Formal analysis, Methodology, Supervision, Validation, Writing – review & editing. LM: Investigation, Methodology, Software, Writing – review & editing. KB: Formal analysis, Methodology, Supervision, Writing – review & editing. SB: Formal analysis, Validation, Writing – review & editing, Funding acquisition. PG: Formal analysis, Validation, Writing – review & editing. AK: Formal analysis, Validation, Writing – review & editing, Funding acquisition. CR: Formal analysis, Funding acquisition, Supervision, Validation, Writing – review & editing.
