# Corrigendum: Gestational hypothyroxinemia induces ASD-like phenotypes in behavior, proinflammatory markers, and glutamatergic protein expression in mouse offspring of both sexes

**DOI:** 10.3389/fendo.2024.1527177

**Published:** 2024-12-12

**Authors:** Enrique González-Madrid, Ma. Andreina Rangel-Ramírez, María C. Opazo, Luis Méndez, Karen Bohmwald, Susan M. Bueno, Pablo A. González, Alexis M. Kalergis, Claudia A. Riedel

**Affiliations:** ^1^ Laboratorio de Endocrino-inmunología, Departamento de Ciencias Biológicas, Facultad de Ciencias de la Vida, Universidad Andrés Bello, Santiago, Chile; ^2^ Millennium Institute on Immunology and Immunotherapy, Facultad de Ciencias Biológicas, Pontificia Universidad Católica de Chile, Santiago, Chile; ^3^ Facultad de Medicina Veterinaria y Agronomía, Instituto de Ciencias Naturales, Universidad de las Américas, Santiago, Chile; ^4^ Instituto de Ciencias Biomédicas, Facultad de Ciencias de la Salud, Universidad Autónoma de Chile, Santiago, Chile; ^5^ Facultad de Ciencias Biológicas, Pontificia Universidad Católica de Chile, Santiago, Chile; ^6^ Departamento de Endocrinología, Facultad de Medicina, Pontificia Universidad Católica de Chile, Santiago, Chile

**Keywords:** prenatal thyroid function, gestational hypothyroxinemia, neurodevelopment, autism spectrum disorder, behavior, inflammation, NLGN3 and HOMER1 expression

In the published article, there was an error in **Figure 10** as published. There are three errors in this figure: 1) The photo for the western blot of tubulin in Figure 10A is repeated in Figure 10C. The correct one is the tubulin in Figure 10A, and the incorrect one is the tubulin photo in Figure 10C. 2) The photo for the western blot of neuroligin in the hippocampus in Figure 10C is repeated with the photo of Homer-1 in the hippocampus in Figure 10G. The correct one is the neuroligin of Figure 10C, and the incorrect one is Figure 10G. 3) The photo for the western blot of tubulin in Figure 10E is repeated with tubulin in Figure 10G. The correct one is in Figure 10E, and the incorrect one is in the photo of Figure 10G. The corrected Figure 10 and its caption appear below.

**Figure 10:**
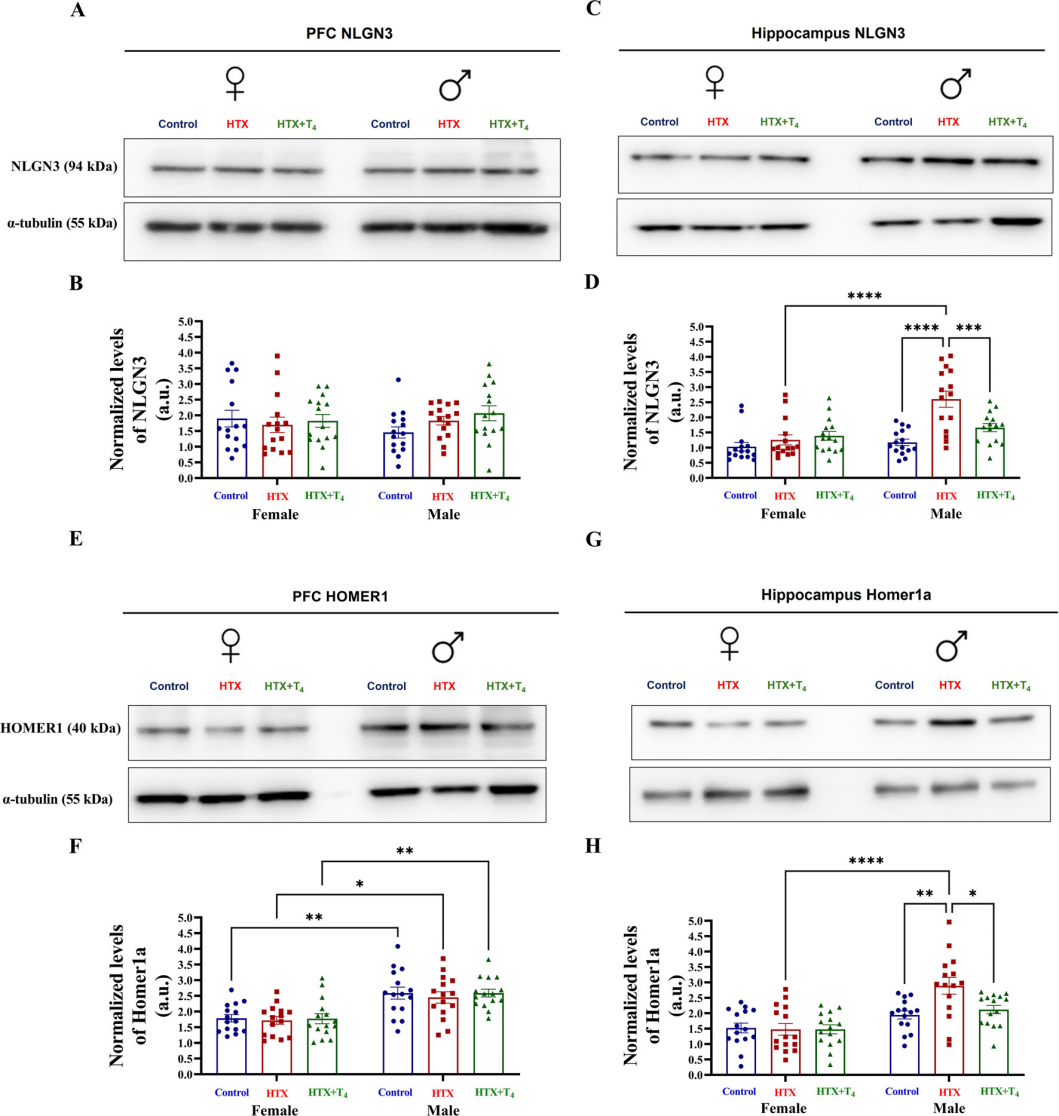
The expression of hippocampal NLGN3 and HOMER1 is increased in male HTX-offspring. Progenies from the three experimental groups were euthanized on P65, and prefrontal cortex (PFC) and hippocampus were isolated. Total proteins were extracted from these tissues and the relative expression of Neuroligin 3 (NLGN3) and HOMER1 from the PFC and hippocampus were evaluated by western blot in each experimental group. **(A)** Representative photography of the western blot of NLGN3 from the PFC, **(B)** the graph shows the normalized relative expression of NLGN3 in the PFC, **(C)** representative photography of the western blot of NLGN3 from the hippocampus, **(D)** the graph shows the normalized relative expression of NLGN3 from the hippocampus, **(E)** representative photography of the western blot of HOMER1 from the PFC, **(F)** the graph shows the normalized relative expression of HOMER1 in the PFC, **(G)** representative photography of the western blot of HOMER1 from the hippocampus, and **(H)** the graph shows the normalized relative expression of HOMER1 from the hippocampus. N =15 per group and sex. a.u means arbitrary units. Data are presented as mean ± S.E.M. Multiple comparisons between experimental groups were analyzed by Mixed-effects model and Tukey’s post-hoc. (*p<0.05, **p<0.01, ***p<0.001, ****p<0.0001). Control-offspring: blue circles, HTX-offspring: red squares, and HTX+T^4^-offspring: green triangles.

The authors apologize for this error and state that this does not change the scientific conclusions of the article in any way. The original article has been updated.

