# Supplementary material for: Gestational hypothyroxinemia induces ASD-like phenotypes in behavior, proinflammatory markers, and glutamatergic protein expression in mouse offspring of both sexes
Source: Front Endocrinol (Lausanne). 2024 May 1;15:1381180. doi: 10.3389/fendo.2024.1381180 (PMC11094302; doi:10.3389/fendo.2024.1381180)
Supplement: Supplementary file 1 [file DataSheet_1.docx]

**Supplementary Table 1**

| **Progenies (C57BL/6 mice)** | **Female (♀)** | | | **Male (♂)** | | |
| --- | --- | --- | --- | --- | --- | --- |
|  | ***Control-offspring*** | ***HTX-offspring*** | ***HTX+T4-offspring*** | ***Control-offspring*** | ***HTX-offspring*** | ***HTX+T4-offspring*** |
| **1.1 Mable burying test** | | | | | | |
| %Buried marbles | 82.78 ± 3.28% ******** | 28.88 ± 4.37% | 83.32 ± 3.49% ******** | 73.88 ± 4.37% ******** | 28.33 ± 3,40% | 78.78 ± 4.52% ******** |
| **1.2 Elevated plus maze test** | | | | | | |
| **a.** %Time spent in open arms | 7.13 ± 1.19% ****** | 1.59 ± 0.61% | 9.44 ± 0.81% ******** | 9.03 ± 1.22% ******** | 1.57 ± 0.77% | 10.47 ± 1.06% ******** |
| **b.** N° entries to open arms | 4.26 ± 0.71% ******** | 0.60 ± 0.24% | 4.13 ± 0.45% ******** | 3.53 ± 0.59% ******* | 0.47 ± 0.16% | 4.00 ± 0.40% ******** |
| **c.** %Time spent in closed arms | 70.76 ± 3.26% ****** | 86.87 ± 1.41% | 74.61 ± 3.06% ***** | 75.55 ± 3.13% ******* | 91.94 ± 2.00% | 76.26 ± 2.65% ****** |
| **d.** N° entries to closed arms | 15.00 ± 1.11% | 14.33 ± 1.18% | 14.00 ± 1.66% | 13.3 ± 1.37% | 12.13 ± 1.27% | 13.67 ± 1.42% |
| **1.3 Tube dominance test** | | | | | | |
| **a.** %Wins (Control vs. HTX) | 79.99 ± 10.98% ***** | 19.99 ± 10.86% | - | 83.34 ± 8.35% ******** | 16.66 ± 8.35% | - |
| **b.** %Wins (HTX+T4 vs. HTX) | - | 27.75 ± 12.89% | 69.63 ± 9.99% ***** | - | 14.44 ± 7.54% | 78.90 ± 7.28% ******* |
| **c.** %Wins (Control vs. HTX+T4) | 48.90 ± 7.43% | - | 49.67 ± 6.35% | 51.00 ± 7.38% | - | 56.20 ± 6.83% |
| **1.4 Three-chamber social preference** | | | | | | |
| **“*Stranger 1*” (S1) stage** | | | | | | |
| **a.** %Time spent in nonsocial compartment | 36.23 ± 2.16% | 39.84 ± 2.17% | 27.28 ± 1.96% ****** | 31.64 ± 1.57% ******** | 51.52 ± 2.19% **a** | 27.47 ± 2.81% ******** |
| **b.** N° entries to nonsocial compartment | 10.40 ± 0.63% | 11.87 ± 1.07% | 10.93 ± 0.58% | 8.06 ± 1.02% ******** | 15.13 ± 0.79% | 11.00 ± 0.66% ****** |
| **c.** %Time direct interaction with nonsocial stimulus | 15.63 ± 0.79% | 23.75 ± 1.59% | 16.34 ± 1.55% | 15.79 ± 0.62% ******** | 31.78 ± 1.03% **b** | 17.69 ± 1.88% ******** |
| **d.** %Time spent in social compartment | 54.19 ± 2.07% | 44.43 ± 2.98% | 53.31 ± 2.81% | 55.61 ± 2.46% ******* | 37.93 ± 3.05% | 51.42 ± 2.32% ****** |
| **e.** N° entries to social compartment | 9.93 ± 0.96% | 8.13 ± 0.88% | 11.67 ± 0.83% | 11.60 ± 1.01% | 8.13 ± 0.60% | 10.93 ± 0.61% |
| **f.** %Time direct interaction with social compartment | 31.45 ± 1.05% ******* | 21.43 ± 0.99% | 32.03 ± 1.20% ******** | 32.51 ± 1.38% ******** | 20.46 ± 1.79% | 29.16 ± 1.45% ****** |
| **“*Stranger 2*” (S2) stage** | | | | | | |
| **g.** %Time spent in S1 compartment | 40.79 ± 2.91% | 46.15 ± 3.79% | 36.04 ± 2.33% | 36.22 ± 2.86% | 47.51 ± 2.06% | 38.34 ± 2.64% |
| **h.** N° entries to S1 compartment | 9.20 ± 1,22% | 12.00 ± 1.53% | 11.33 ± 0.90% | 11.00 ± 0.92% ***** | 16.40 ± 1.35% | 10.93 ± 0.87% ***** |
| **i.** %Time direct interaction with S1 compartment | 20.62 ± 1.24% ****** | 27.21 ± 1.20% | 16.01 ± 0.92% ******** | 19.51 ± 0.80% ******** | 28.36 ± 1.17% | 17.44 ± 1.86% ******** |
| **j.** %Time spent in S2 compartment | 51.30 ± 2.57% ***** | 41.44 ± 3.37% | 54.58 ± 1.69% ****** | 53.76 ± 2.18% ******* | 37.05 ± 1.69% | 47.86 ± 1.70% ***** |
| **k.** N° entries to S2 compartment | 12.20 ± 1.40% ***** | 7.93 ± 0.84% | 12.67 ± 0.71% ***** | 13.53 ± 0.80% | 10.13 ± 0.96% | 13.67 ± 1.06% |
| **l.** %Time direct interaction with S2 compartment | 31.81 ± 1.47% ******* | 22.63 ± 1.60% | 30.08 ± 0.55% ****** | 29.38 ± 1.30% ******* | 20.59 ± 1.94% | 31.76 ± 0.80% ******** |

**Supplementary Table 1. Data of behavioral assessment.** This information was obtained from each test implemented to evaluate autism-like behaviors. (**1**) Marble burying test (for repetitive behavior), (**2**) Elevated plus maze (for anxiety), (**3**) Tube dominance test, and (**4**) Three chamber social preference test (for social interaction abilities). These tests were performed in the Control-offspring, HTX-offspring, and HTX+T_4_-offspring. Moreover, we separated the groups based on sex. Measured parameters are defined in the Materials and methods section. N = 15 per experimental group and sex. Data are presented as mean ± S.E.M. Multiple comparisons between experimental groups were analyzed by Mixed-effects model and Tukey’s post-hoc. Analyses were performed using Prism 9.0.2 software. *p* values are presented as follows: **p*<0.05, ***p*<0.01, ****p*<0.001, and *****p*<0.0001. Differences between sexes are indicated by letters. Specifically, ‘**a**’ indicates ****p*<0.001 when comparing male HTX-offspring with female HTX-offspring (item **1.4a** of the table), and ‘**b’** indicates *****p*<0.0001 when comparing male HTX-offspring with female HTX-offspring (item **1.4c** of the table). These differences between sexes are also highlighted in **SF 2A** and **Figure** **5A**, respectively.

**Supplementary Figure 1**

**Supplementary Figure 1. The HTX-offspring has a major preference for nonsocial compartment in the three-chamber social test.** Progenies were subjected to analyze their social interaction skills with the three-chamber social preference test on P64 (see Materials and methods). In the “Stranger 1” step, mice were exposed to interact with a nonsocial and a social stimulus. The following parameters were registered: (**A**) Percentage of time spent in the nonsocial compartment, (**B**) number of entrances to the nonsocial compartment, (**C**) Percentage of time spent in the social compartment, and (**D**) number of entrances to the social compartment. N = 15 per group and sex. Data are presented as mean ± S.E.M. Multiple comparisons between experimental groups and sexes were analyzed by Mixed-effects model and Tukey’s post-hoc. (**p*<0.05, ***p*<0.01, ****p*<0,001, *****p*<0.0001). Control-offspring: blue circles, HTX-offspring: red squares, and HTX+T_4_-offspring: green triangles.

**Supplementary Figure 2**

**Supplementary Figure 2. The HTX-offspring exhibit a less preference for the novel stimuli in the Three-chamber social preference test.** Progenies were subjected to analyze their social interaction skills with the three-chamber social preference test on P64 (see Materials and methods). In the “Stranger 2” step, mice were exposed to interact with the social stimulus from the “Stranger 1” step and with a new social incentive (“Stranger 2”). The following parameters were registered: (**A**) Percentage of time spending in the S1 compartment, (**B**) number of entrances to the S1 compartment, (**C**) Percentage of time spending in the S2 compartment, and (**D**) number of entrances to the S2 compartment. N = 15 per group and sex. Data are presented as mean ± S.E.M. Multiple comparisons between experimental groups and sexes were analyzed by Mixed-effects model and Tukey’s post-hoc. (**p*<0,05, ***p*<0.01, ****p*<0,001, *****p*<0.0001). Control-offspring: blue circles, HTX-offspring: red squares, and HTX+T_4_-offspring: green triangles.

**Supplementary Table 2**

| **Progenies (C57BL/6 mice)** | **Female (♀)** | | | **Male (♂)** | | |
| --- | --- | --- | --- | --- | --- | --- |
|  | ***Control-offspring*** | ***HTX-offspring*** | ***HTX+T4-offspring*** | ***Control-offspring*** | ***HTX-offspring*** | ***HTX+T4-offspring*** |
| **2.1. Cytokines determination in serum**  **samples (data normalized to volume of serum samples) (pg/mL)** | | | | | | |
| **a.** TNF-α | 77.41 ± 10.75 ******** | 232.08 ± 15.27 | 44.47 ± 5.47 ******** | 67.59 ± 8.37 ******** | 233.43 ± 12.73 | 49.73 ± 6.34 ******** |
| **b.** IL-6 | 52.26 ± 6.05 ******* | 123.12 ± 14.64 | 36.52 ± 4.98 ******** | 49.47 ± 6.85 ******** | 127.91 ± 15.30 | 52.42 ± 10.92 ******** |
| **c.** IL-1β | 41.20 ± 5.55 ******** | 159.57 ± 15.75 | 31.87 ± 4.21 ******** | 39.93 ± 5.72 ******** | 206.36 ± 14.35 **a** | 32.93 ± 3.25 ******** |
| **d.** IL-17A | 26.47 ± 2.37 ******** | 247.60 ± 16.71 | 32.87 ± 4.66 ******** | 25.47 ± 23,34 ******** | 246.60 ± 21.13 | 25.27 ± 2.15 ******** |
| **e.** IL-10 | 35.20 ± 2.73 | 22.67 ± 3.41 | 33.93 ± 4.09 | 38.87 ± 3.99 ******** | 12.60 ± 2.14 | 40.73 ± 3.32 ******** |
| **2.2. Cytokines determination in brain portions (data normalized to 0.5 mg of proteins) (pg/mg)** | | | | | | |
| **Prefrontal cortex (PFC)** | | | | | | |
| **a.** IL-17A | 0.107 ± 0.005 ***** | 0.140 ± 0.013 | 0.106 ± 0.007 ***** | 0.118 ± 0.007 ******* | 0.170 ± 0.005 | 0.123 ± 0.007 ****** |
| **b.** IL-10 | 0.023 ± 0.002 | 0.027 ± 0.004 | 0.028 ± 0.003 | 0.038 ± 0.004 ****^,^ b** | 0.017 ± 0.003 | 0.039 ± 0.004 ******* |
| **Hippocampus** | | | | | | |
| **c.** IL-17A | 0.111 ± 0.009 ******** | 0.161 ± 0.006 | 0.121 ± 0.004 ******* | 0.131 ± 0.009 ****** | 0.170 ± 0.006 | 0.124 ± 0.005 ******* |
| **d.** IL-10 | 0.036 ± 0.005 | 0.025 ± 0.004 | 0.033 ± 0.003 | 0.034 ± 0.005 ***** | 0.015 ± 0.002 | 0.036 ± 0.003 ****** |

**Supplementary Table 2.** **Cytokines determination.** Mice were euthanized after behavioral assessment and blood was extracted by cardiac puncture. (**1**) Serum was isolated from each mouse and cytokines were quantified in samples of 100 μL. (**2**) In parallel, brains were also removed, and the prefrontal cortex (PFC) and hippocampus were dissected. Total proteins were extracted from both tissues, quantified, and 0.5 mg were used as samples for cytokines determination (see Materials and methods). N = 15 per experimental group and sex. Data are presented as mean ± S.E.M. Multiple comparisons between experimental groups were analyzed by Mixed-effects model and Tukey’s post-hoc. Analyses were performed using Prism 9.0.2 software. *p* values are presented as follows: **p*<0.05, ***p*<0.01, ****p*<0.001, and *****p*<0.0001. Differences between sexes are indicated by letters. Specifically, **‘a’** indicates **p*≤0.05 when comparing male HTX-offspring with female HTX-offspring (item **4.1c** of the table), and **‘b’** to indicate **p*≤0.05 when comparing male Control-offspring with female Control-offspring (item **4.2b** of the table). These differences between sexes are also highlighted in **Figure 6C** and **7B**, respectively.

**Supplementary Figure 3**

**A**


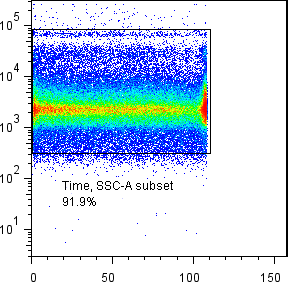

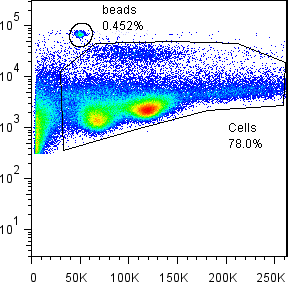

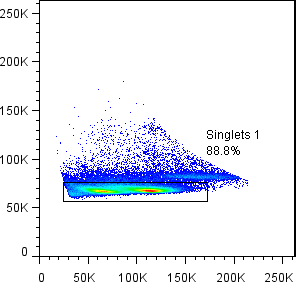

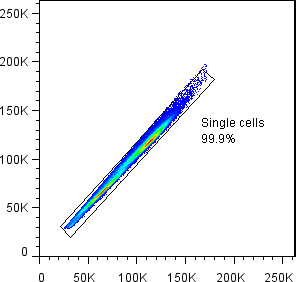
**Time Autofluorescence Singlets 1 Singlets 2**

# B

**Live cells**


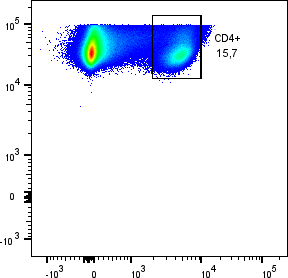


SSC-A

SSC-A


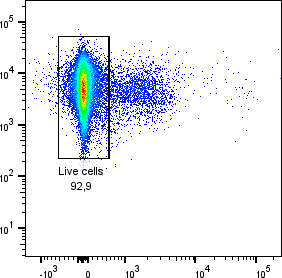


AF700 CD4 – BUV395

IL-17A – APCCy7

CD25 – PeCy7

CD4 – BUV395

FOXP3 - Pe


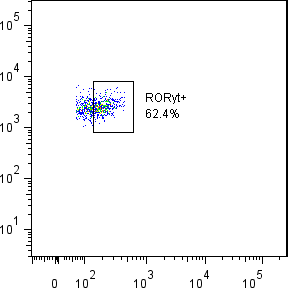


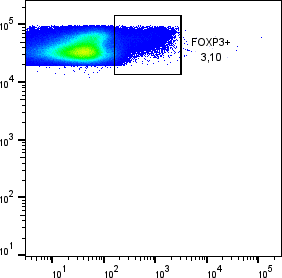
RORγt – BV421


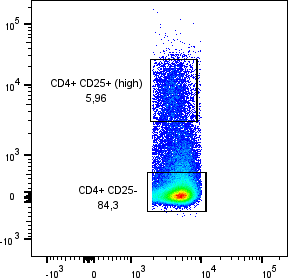


CD4 – BUV395


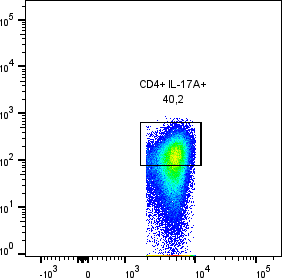


SSC-A

SSC-A

**CD4+CD25highFOXP3+ cells (Tregs)**

## CD4+IL-17+RORγt+ cells (Th17)

**Supplementary Figure 3. Gating strategy for Tregs and Th17 cells detection.** (**A**) General selection from Time control to Single cells. (**B**) Selection of Treg cells and Th17 from Live cells (negative events for AF700). CD4-positive events were gated, and FOXP3-positive events were detected inside the double positive selection of CD4^+^CD25^(high)^. CD4^+^CD25^(high)^FOXP3^+^ population was considered as Treg cells in this work. In parallel, a double-positive events for CD4^+^ and IL-17A^+^ were gated from the CD4^+^CD25^-^ population. RORγt-positive events from the previous CD4^+^IL-17A^+^ population were gated. CD4^+^IL-17A^+^ RORγt^+^ population was considered as Th17 cells in this work.

**Supplementary Figure 4**

**A**

**Time Autofluorescence Singlets 1 Singlets 2**


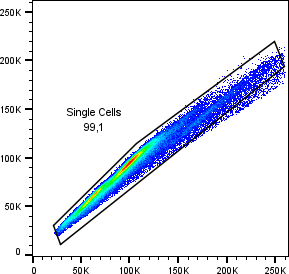

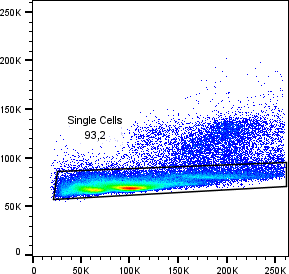

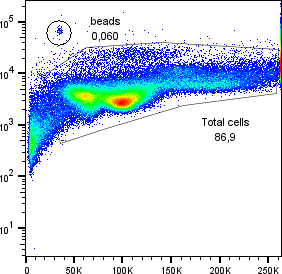

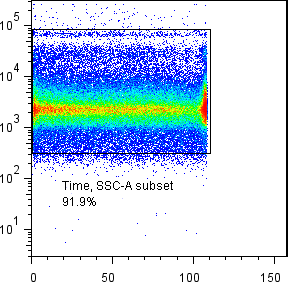


**B**

**(M1-type macrophages)**


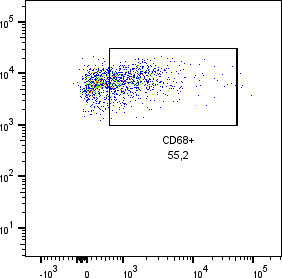
**Live cells**


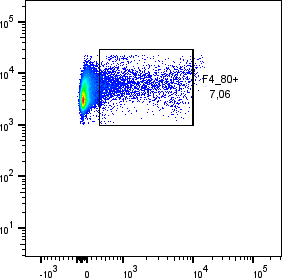

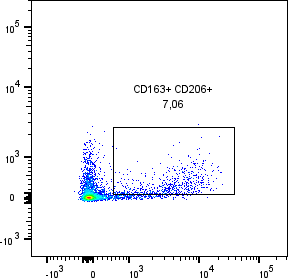

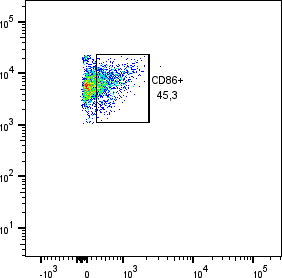


SSC-A

CD3 – PercpCy5.5

SSC-A

SSC-A

SSC-A

SSC-A


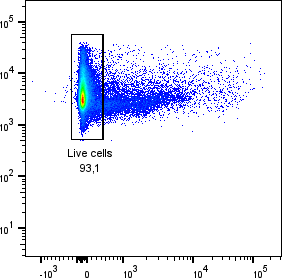

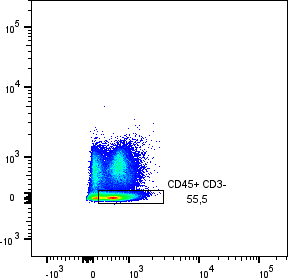


AF700 CD45 – FITC

F4/80 – BUV395

CD80 – BV605

CD68 – PeCy7

###

CD206 – BV421

**(M2-type macrophages)**

CD163 - APC

#
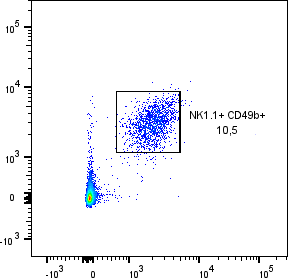
C

CD49b - APC

**Live cells**


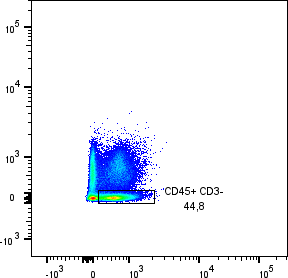


SSC-A

CD3 – PercpCy5.5


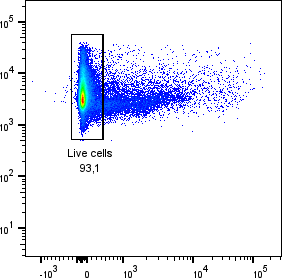


AF700 CD45 – FITC

CD11b – PeCy7


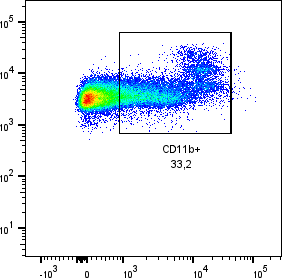


SSC-A

NK1.1 – BV605

**(NK cells)**

**Supplementary Figure 4**. **Gating strategy for M1/M2-type macrophages and NKs detection.** (**A**) General selection from Time control gate to Single cells. (**B**) Selection of M1/M2-type macrophages from Live cells (total negative events for AF700). CD45-positive/CD3-negative population was selected, and F4/80-positive events from that population were gated. CD80-positive events followed by the CD68-positive population were gated to define the M1-type macrophage phenotype: CD45^+^CD3^-^F4/80^+^CD80^+^CD68^+^ cells. In parallel, the double-positive CD163/CD206 population was selected from the F4/80-positive gate to define the M2-type macrophage phenotype: CD45^+^CD3^-^F4/80^+^CD163^+^CD206^+^ cells. (**C**) NK cells were detected from Live cells (total negative events for AF700). CD45-positive/CD3-negative population was gated, and CD11b-positive events from that selection were gated. Finally, the double-positive NK1.1/CD49b population was selected from the previous gate to define the NK phenotype: CD45^+^CD3^-^CD11b^+^NK1.1^+^CD49b^+^ cells.

**Supplementary Figure 5**

**Supplementary Figure 5. Levels of total thyroid hormones remained consistent among Control, HTX, and HTX+T_4_ offspring.** Levels of total TH and TSH were measured in blood samples obtained from the facial vein of Control, HTX, and HTX+T_4_ offspring on P50 and plotted at (**A**) tT_4_, (**B**) tT_3_, and (**C**) TSH. Data is shown combining females and males for each experimental group. N=30 per group. Data are presented as mean ± S.E.M. Differences were analyzed by *one-way* ANOVA and Tukey’s post-hoc. Control-offspring: blue circles, HTX-offspring: red squares, and HTX+T_4_-offspring: green triangles.

**Supplementary Figure 6**

**Supplementary Figure 6. The HTX-gestated offspring buried fewer marbles then the Control and HTX+T_4_ offspring.** To assess repetitive behavior, adult offspring from the three experimental groups underwent the marble burying test on P55. The number of buried marbles was recorded, and the average count per experimental group was calculated. Data is shown combining females and males for each experimental group. N=30 per group. Data are presented as mean ± S.E.M. Differences were analyzed by *one-way* ANOVA and Tukey’s post-hoc. (*****p*< 0.0001). Control-offspring: blue circles, HTX-offspring: red squares, and HTX+T_4_-offspring: green triangles.

**Supplementary Figure 7**

**Supplementary Figure 7. The HTX-gestated offspring exhibited anxious-like behavior.** To assess anxious-like behavior, adult offspring from the three experimental groups were subjected to the elevated plus maze test on P56. (**A**) Percentage of time spent in open arms, (**B**) Number of entrances into the open arms, (**C**) Percentage of time spent in closed arms, and (**D**) Number of entrances into the closed arms. Data is shown combining females and males for each experimental group. N=30 per group. Data are presented as mean ± S.E.M. Differences were analyzed by *one-way* ANOVA and Tukey’s post-hoc. (*****p*< 0.0001). Control-offspring: blue circles, HTX-offspring: red squares, and HTX+T_4_-offspring: green triangles.

**Supplementary Figure 8**

**Supplementary Figure 8. The HTX-gestated offspring displayed a subordinate state in the tube dominance test.** Offspring from the three experimental groups were submitted to the tube dominance test (from P57 to P63) to study their socially aggressive behavior. Confrontations were arranged as follows: (**A**) Control-offspring vs. HTX-offspring, (**B**) HTX-offspring vs. HTX+T_4_-offspring, and (**C**) Control-offspring vs. HTX+T_4_-offspring. The percentage of wins was plotted. Data is shown combining females and males for each experimental group. N=30 per group. Data are presented as mean ± S.E.M. Differences were analyzed by *one-way* ANOVA and Tukey’s post-hoc. (*****p*<0.0001). Control-offspring: blue circles, HTX-offspring: red squares, and HTX+T_4_-offspring: green triangles.

**Supplementary Figure 9**

**Supplementary Figure 9. The HTX-gestated offspring displayed a less direct social interaction preference to unknown individuals compared to Control and HTX+T_4_ offspring.** Offspring from the three experimental groups were submitted to analyze their social interaction abilities with the three-chamber social preference test on P64. In the “Stranger 1” step, mice were exposed to a nonsocial and a social stimulus. (**A**) Percentage of time of direct interaction with the nonsocial stimulus and (**B**) Percentage of time of direct interaction with the social stimulus. In the “Stranger 2” step, mice were exposed to interact with the social stimulus from the first step but now renamed as “Stranger 1” step and the nonsocial stimuli was replaced with a new social incentive named “Stranger 2”. (**C**) Percentage of time of direct interaction with the S1 stimulus, and (**D**) Percentage of time of direct interaction with the S2 stimulus. Data is shown combining females and males for each experimental group. N=30 per group. Data are presented as mean ± S.E.M. Differences were analyzed by *one-way* ANOVA and Tukey’s post-hoc. (**p*<0.05, *****p*< 0.0001). Control-offspring: blue circles, HTX-offspring: red squares, and HTX+T_4_-offspring: green triangles.

**Supplementary Figure 10**

**Supplementary Figure 10. The HTX-gestated offspring has an increased concentration of pro-inflammatory cytokines in blood, while only male showed a reduced concentration of IL-10.** Control, HTX, and HTX+T_4_  offspring were euthanized on P65 and the blood was extracted by cardiac puncture. The serum was isolated and used as samples (100 μL each) for the following cytokines determination by using sandwich ELISA: (**A**) TNF-α, (**B**) IL-6, (**C**) IL-1β, (**D**) IL-17A, and (**E**) IL- 10. Data is shown combining females and males for each experimental group. N=30 per group. Data are presented as mean ± S.E.M. Differences were analyzed by *one-way* ANOVA and Tukey’s post-hoc. (**p*<0.05, *****p*<0.0001). Control-offspring: blue circles, HTX-offspring: red squares, and HTX+T_4_-offspring: green triangles.

**Supplementary Figure 11**

**Supplementary Figure 11. The HTX-gestated offspring has increased levels of IL-17A in the prefrontal cortex and hippocampus and reduced IL-10 levels.** Control, HTX, and HTX+T_4_  offspring were euthanized on P65 and PFC and hippocampus were isolated. Total proteins were extracted from these tissues and 0.5 mg per sample were used for the measurement of IL-17A and IL-10 by sandwich ELISA. (**A**) IL-17A in the PFC, (**B**) IL-10 in the PFC, (**C**) IL-17A in the hippocampus, and (**D**) IL-10 in the hippocampus. Data was normalized to mg of proteins. Data is shown combining females and males for each experimental group. N=30 per group. Data are presented as mean ± S.E.M. Differences were analyzed by *one-way* ANOVA and Tukey’s post-hoc. (***p*<0.01, ****p*<0,001, *****p*<0.0001). Control-offspring: blue circles, HTX-offspring: red squares, and HTX+T_4_-offspring: green triangles.

**Supplementary Figure 12**

**Supplementary Figure 12. The HTX-gestated offspring has increased Th17/Tregs ratio compared to Control and HTX+T_4_ offspring.** Progenies from the three experimental groups were euthanized on P65, and spleens were removed. Total splenocytes were purified and a portion of these cells was *in vitro* stimulated using PMA-Ionomycin-BFA. The population of Tregs and Th17 were quantified by flow cytometry. (**A**) Absolute number of FOXP3-positive cells (Treg cells), (**B**) absolute number of RORγt-positive cells (Th17 cells), and (**C**) the graph shows the Th17/Tregs ratio. Data is shown combining females and males for each experimental group. N=30 per group. Data are presented as mean ± S.E.M. Differences were analyzed by *one-way* ANOVA and Tukey’s post-hoc. (*****p*<0.0001). Control-offspring: blue circles, HTX-offspring: red squares, and HTX+T_4_-offspring: green triangles.

**Supplementary Figure 13**

**Supplementary Figure 13. M1-type macrophages are increased in HTX-offspring’s spleen, while M2-type macrophages and NK cells remain similar among experimental groups.** Splenic innate immune cells were quantified by flow cytometry. (**A**) Absolute number of CD68-positive cells (M1-like macrophages), (**B**) absolute number of CD206/CD163 double-positive cells (M2-like macrophages), and (**C**) absolute number of NK1.1/CD49b double-positive cells (NK cells). Data is shown combining females and males for each experimental group. N=30 per group. Data are presented as mean ± S.E.M. Differences were analyzed by *one-way* ANOVA and Tukey’s post-hoc. (**p*<0.05, *****p*<0.0001). Control-offspring: blue circles, HTX-offspring: red squares, and HTX+T_4_-offspring: green triangles.

**Supplementary Figure 14**

**Supplementary Figure 14. The expression levels of hippocampal are increased in the HTX-offspring.** Progenies from the three experimental groups were euthanized on P65, and PFC and hippocampus were isolated. Total proteins were extracted from these tissues and the relative expression of Neuroligin 3 (NLGN3) and HOMER1 from the PFC and hippocampus were evaluated by western blot in each experimental group. (**A**) Normalized relative expression of NLGN3 in the PFC, (**B**) normalized relative expression of NLGN3 from the hippocampus, (**C**) normalized relative expression of HOMER1 in the PFC, and (**D**) normalized relative expression of HOMER1 from the hippocampus. Data is shown combining females and males for each experimental group. N=30 per group. Data are presented as mean ± S.E.M. Differences were analyzed by *one-way* ANOVA and Tukey’s post-hoc. (****p*<0.001). Control-offspring: blue circles, HTX-offspring: red squares, and HTX+T_4_-offspring: green triangles.

**Supplementary Figure 15**

**A**

**B**

| **Parameter** | **Correlated phenotypes in offspring** | ***r*** | ***p* value** |
| --- | --- | --- | --- |
| %Buried Marbles | N° entries to open arms | 0,5837498 | *p*<0,00001 |
|  | Direct interaction social stimuli | 0,6032614 |  |
|  | IL-1β in serum | -0,580006 |  |
|  | IL-17A in serum | -0,6564256 |  |
|  | TNF-α in serum | -0,5723387 |  |
|  | IL-17A in hippocampus | -0,6395581 |  |
|  | Ratio Th17/Treg | -0,6034223 |  |
| Time spent in open arms | N° entries to open arms | 0,6025257 | *p*<0,00001 |
|  | Time spent in closed arms | -0,570077 |  |
|  | IL-6 in serum | -0,5983087 |  |
|  | IL-17A in serum | -0,5735446 |  |
|  | TNF-α in serum | -0,6720549 |  |
| N° entries to open arms | Time spent in closed arms | -0,580821 | *p*<0,00001 |
|  | Direct interaction S1 stimuli | -0,5748609 |  |
|  | IL-1β in serum | -0,6006288 |  |
|  | IL-17A in serum | -0,6419249 |  |
|  | TNF-α in serum | -0,5980513 |  |
|  | M1-like macrophages | -0,5565098 |  |
| Time spent in closed arms | TNF-α in serum | 0,5539875 | *p*<0,00001 |
| Direct interaction social stimuli | IL-1β in serum | -0,5869915 | *p*<0,00001 |
|  | IL-17A in serum | -0,5670174 |  |
|  | TNF-α in serum | -0,5603076 |  |
| Direct interaction S1 stimuli | IL-1β in serum | 0,653406 | *p*<0,00001 |
|  | IL-6 in serum | 0,5586518 |  |
|  | TNF-α in serum | 0,6378314 |  |
|  | M1-like macrophages | 0,6669249 | 0,00171 |
| IL-1β in serum | IL-17A in serum | 0,6319126 | *p*<0,00001 |
|  | TNF-α in serum | 0,6488574 |  |
|  | M1-like macrophages | 0,5885768 |  |
| IL-6 in serum | TNF-α in serum | 0,614042 | *p*<0,00001 |
|  | M1-like macrophages | 0,5828384 |  |
| IL-17A in serum | TNF-α in serum | 0,6683716 | *p*<0,00001 |
|  | M1-like macrophages | 0,6359658 |  |
| TNF-α in serum | IL-17A in PFC | 0,6338232 | *p*<0,00001 |
|  | M1-like macrophages | 0,6306602 |  |

**Supplementary Figure 15. Pearson correlation analysis between all the observed outcomes in the offspring.** Using all data obtained from mice within each experimental group, comprehensive Pearson correlation analysis was conducted. (**A**) Correlation matrix showing full spectrum of identified associations. (**B**) Summarized table highlighting all moderate-to-strong associations, suggesting the correlation between behavior alterations with proinflammatory traits in the HTX-offspring.
